# Domain Wall Dynamics in a Ferroelastic Spin Crossover Complex with Giant Magnetoelectric Coupling

**DOI:** 10.1021/jacs.1c08214

**Published:** 2021-12-23

**Authors:** Vibe Boel Jakobsen, Elzbieta Trzop, Emiel Dobbelaar, Laurence C. Gavin, Shalinee Chikara, Xiaxin Ding, Minseong Lee, Kane Esien, Helge Müller-Bunz, Solveig Felton, Eric Collet, Michael A. Carpenter, Vivien S. Zapf, Grace G. Morgan

**Affiliations:** † School of Chemistry, University College Dublin, Belfield, Dublin 4, Ireland; ‡ Univ Rennes, CNRS, IPR (Institut de Physique de Rennes)-UMR 6251, F-35000 Rennes, France; § Department of Physics, Auburn University Auburn, Alabama 36849, United States; || National High Magnetic Field Laboratory, Los Alamos National Laboratory, Los Alamos, New Mexico 87545, United States; ⊥ Centre for Nanostructured Media, School of Mathematics and Physics, Queen’s University of Belfast, Belfast BT7 1NN, Northern Ireland, United Kingdom; # Department of Earth Sciences, University of Cambridge, Downing Street, Cambridge CB2 3EQ, England, United Kingdom

## Abstract

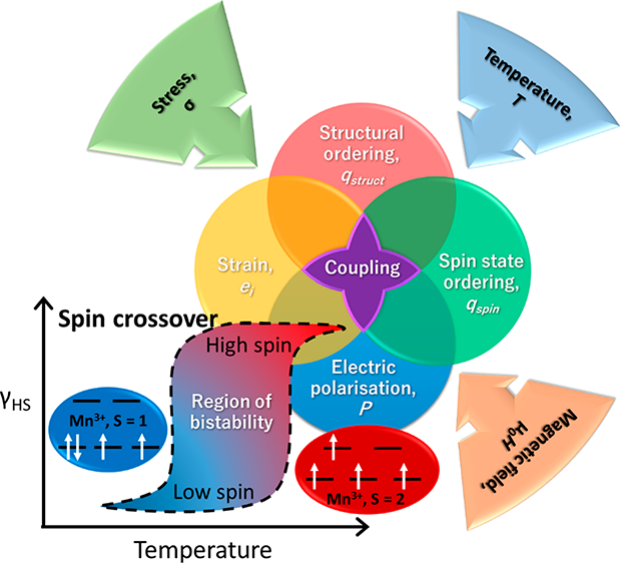

Pinned and mobile ferroelastic domain walls are detected in response to mechanical stress in a Mn^3+^ complex with two-step thermal switching between the spin triplet and spin quintet forms. Single-crystal X-ray diffraction and resonant ultrasound spectroscopy on [Mn^III^(3,5-diCl-sal_2_(323))]BPh_4_ reveal three distinct symmetry-breaking phase transitions in the polar space group series *Cc* → *Pc* → *P*1 → *P*1_(1/2)_*.* The transition mechanisms involve coupling between structural and spin state order parameters, and the three transitions are Landau tricritical, first order, and first order, respectively. The two first-order phase transitions also show changes in magnetic properties and spin state ordering in the Jahn–Teller-active Mn^3+^ complex. On the basis of the change in symmetry from that of the parent structure, *Cc*, the triclinic phases are also ferroelastic, which has been confirmed by resonant ultrasound spectroscopy. Measurements of magnetoelectric coupling revealed significant changes in electric polarization at both the *Pc* → *P*1 and *P*1 → *P*1_(1/2)_ transitions, with opposite signs. All these phases are polar, while *P*1 is also chiral. Remanent electric polarization was detected when applying a pulsed magnetic field of 60 T in the *P*1→ *P*1_(1/2)_ region of bistability at 90 K. Thus, we showcase here a rare example of multifunctionality in a spin crossover material where the strain and polarization tensors and structural and spin state order parameters are strongly coupled.

## Introduction

Domain wall engineering^1,2^ in ferroic materials (ferromagnets, ferroelectrics, ferroelastics) constitutes one of the most promising areas for new applications in nanoelectronics^3^ and nanomagnetism.^4^ It is now well-established that the properties which emerge within domain walls are distinct from the properties within the domains themselves, and thus domain walls are recognized as functional 2D objects in their own right, rather than mere barriers between ordered functional regions. Examples of novel properties include conductivity^5−7^ or superconductivity^8^ in the domain walls of insulating ferroelectrics, ferromagnetic ordering of ferroelectric antiferromagnets,^3^ and unexpected photovoltaic effects concentrated in the domain walls of ferroelectrics where the voltage is higher than the band gap of the parent material.^9−11^ The mobility of domain walls allows them to be moved by electric or magnetic fields or by stress with velocities dependent on the wall dimensions and scale of the driving field. For example, the concept of using current driven data storage in magnetic racetrack memory devices via the supersonic motion of magnetic domain walls, which tend to be relatively broad (tens of nanometers), is well advanced.^12,13^ On the other hand, the potential for dynamic circuitry in ferroelectrics, where domain walls have lower velocities and are typically more narrow (widths of the order of one unit cell), is only starting to be realized.^14−16^ Memory devices based on the wall properties of ferroelectrics therefore have the potential for higher density storage that will outperform the bulk material, as the higher number of narrow domain walls means they constitute around 1% of the material by total volume.^17,18^

Phase transitions within a domain wall, as opposed to those in the bulk material, may also confer new functionality. This is typified in some nonpolar ferroelastic perovskite oxides such as SrTiO_3_, where thermal-induced polarity appears at cryogenic temperatures, indicative of a two-dimensional phase transition within the ferroelastic twin walls that are atomistically thin.^19−22^ The emergence of new order parameters during a move through a phase transition can thus lead to “domains with domains and walls within walls”,^19^ and materials with such multicomponent order parameters should offer both novel functionality and associated switching opportunities.

An investigation of the dimensions and mobility achievable in domain walls of many different types of materials is required to determine the switching speed and power efficiency, which in turn will inform the design process for potential device applications. In this respect it is of interest to look beyond oxide physics and investigate the ferroic and multiferroic potential of molecule-based systems, where the greater scope for changes in the degrees of freedom of both translational and point group symmetries may yield multiple order parameters over a phase transition or phase transitions. In particular, the large atomic displacements and resulting volume change associated with spin state switching in spin crossover (SCO) transition-metal complexes^23−28^ should ensure a change in strain gradient, especially for those systems with sharp transitions. Thermal SCO is often accompanied by structural phase transitions^29−47^ and associated spin state ordering, a phenomenon which can be classified as ferroic and which may be periodic^27,48,49^ or aperiodic.^50^

As highlighted in recent reviews, a strong cooperative effect occurs when SCO gives rise to a sequence of phase transitions and hysteretic SCO behavior.^27,48,49^ The diversity of possible ordering schemes for HS and LS cations, for example, has been simulated for a 60 × 60 lattice by Cruddas and Powell.^51^ A formalism from Landau theory provides a convenient phenomenological means of analyzing this behavior in terms of elastic coupling between the order parameter associated with SCO and symmetry-breaking order parameters due to ordering of HS and LS atoms.^52,53^ In the limiting case of SCO occurring without any change in crystallographic symmetry, the evolution of a single order parameter monitoring the global evolution of the spin state is sufficient to characterize the evolution of properties such as magnetic susceptibility, unit cell volume, and elastic moduli. This parameter has the symmetry of the identity representation of the space group and is therefore a non-symmetry-breaking order parameter *q*_spin_, as it preserves spatial symmetry.^54,55^ Symmetry-breaking order parameters are required to define the ordered structures and will have, in the space group of the parent (HS) high-symmetry structure, the symmetry of the irreducible representation which is related to the space group of the ordered structure (HS+LS). More generally, the non-symmetry-breaking spin state order parameter can couple with the order parameter for any structural instability, leading to many possibilities for the influence of SCO in multiferroic materials.

If there is coupling between the spin state and structural order parameters, there must also be coupling of their gradients through domain walls. The emergence of multicomponent order parameters can thus be expected to give rise to a diversity of domain wall properties and behaviors in spin state ordered crystals at low temperatures. We have recently reported the use of resonant ultrasound spectroscopy (RUS) to detect stress-induced ferroelastic domain wall motion in a Mn^3+^ SCO complex where ferroelastic and spin state order parameters are coupled.^30^ The mobile domain walls are distinct from the expected mobile phase boundary that develops at the high spin/low spin (HS/LS) boundary in isostructural SCO systems, where there is no change in structural order parameter and which can be followed effectively by optical microscopy.^56−66^ We now employ RUS to follow the ferroelastic changes that accompany the two-step thermal SCO in a Mn^3+^ complex which reveals both mobile and pinned domain walls at the phase transitions.

SCO in Mn^3+^ systems may be particularly useful in ferroelastic applications, as the Jahn–Teller effect in the spin quintet form results in a strong and thermally switchable distortion that may enhance the spin–lattice coupling. If this results in a phase transition that leads to broken inversion symmetry, a resulting magnetoelectric (ME) coupling may be observed.^67−74^ The expectation must be that complexes which exhibit spin–lattice coupling will result in the development of SCO transitions that give rise to strong multiferroic coupling behavior because of the possibility of common strain providing a coupling mechanism among magnetic, ferroelectric, and ferroelastic properties. This has been extensively studied in metal oxides but has rarely been reported in SCO materials.^67−74^ One reported example is Mn^3+^ in the SCO complex Mn(taa) (H_3_taa = tris(1-(2-azolyl)-2-azabuten-4-yl)amine), which undergoes a magnetic-field-induced SCO between *S* = 1 and *S* = 2 states above 35 T. A C3 → C1 Jahn–Teller effect induces electric dipoles in the *S* = 2 state that order to create electric polarization.

**Scheme 1:**
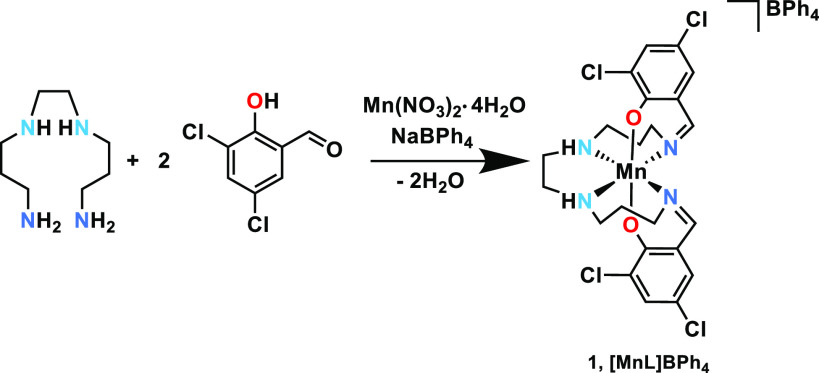
Synthesis of Complex **1**, [Mn(3,5-diCl-sal_2_(323))]BPh_4_

We now report hysteretic two-step SCO behavior in complex **1**, [Mn(3,5-diCl-sal_2_(323))]BPh_4_ (Scheme 1), which is accompanied by structural phase transitions between polar space groups with different electric polarizations, and we also demonstrate the existence of both mobile and pinned domain walls at low temperature. We have investigated four structural phases that occur in **1** at different temperatures via single-crystal X-ray diffraction and RUS. On cooling from room temperature, the full transition sequence in terms of space groups is *Cc* → *Pc* → *P*1 → *P*1_(1/2)_. The four distinct crystallographic phases are consistent with spin state ordering stemming from the different HS and LS populations in the sequence **Cc*[HS] →*Pc*[HS:HS]*→ P**1 *[LS:LS:HS:HS] → P*1 [LS:LS] . Elastic anomalies due to coupling of strain with the structural and spin state order parameters have been analyzed by RUS, while coupling between the spin state order parameter and electric polarization was determined from pulsed and DC magnetic field magnetization and electric polarization data. Complex **1** displays a strong magnetoelectric effect in single-crystal form.

## Results and Discussion

### Two-Step Thermal Spin Transition

**Figure 1:**
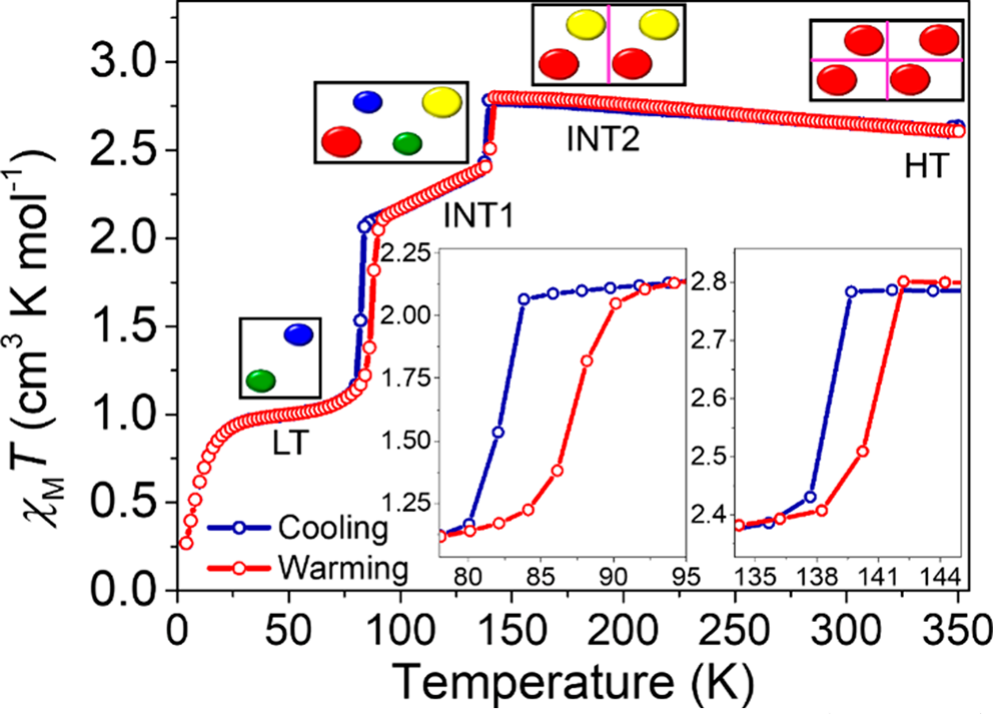
χ_M_*T* versus *T* for complex **1** measured in cooling (blue curve) and warming (red curve) modes between 4 and 350 K with an applied magnetic field, μ_0_*H*, of 0.1 T. The insets show the 3 K wide hysteretic transition taken from the midpoints of the transition from 83 to 86 K and the 1 K hysteretic transition from 139 to 140 K. Four crystallographically distinct phases were detected by single-crystal X-ray diffraction (*vide infra*), and each unit cell is represented by the black box with the unit cell contents depicted with colored ellipses. The large red and yellow ellipses represent Mn^3+^ sites in the *S* = 2 HS state, and the small blue and green ellipses represent Mn^3+^ sites in the *S* = 1 LS state. Pink lines represent a simplified view of symmetry elements present in the unit cell.

The temperature-dependent magnetic susceptibility (χ_M_*T*) between 4 and 350 K revealed a two-step thermal SCO in complex **1**. As shown in Figure 1, a χ_M_*T* value of 2.60 cm^3^ K mol^–1^ was observed at 350 K, which is close to the value of 3.0 cm^3^ K mol^–1^ expected for *S* = 2 from the spin-only formula (with *g* = 2).^29,30,75−79^ The χ_M_*T* value increases slightly to 2.80 cm^3^ K mol^–1^ on cooling to 140 K (intermediate phase 2, INT2) and then abruptly drops to 2.39 cm^3^ K mol^–1^ around 135 K, with the transition temperature *T*_1/2,INT1_↓= 139 K (intermediate phase 1, INT1). Upon further cooling, the χ_M_*T* value decreases gradually to 2.07 cm^3^ K mol^–1^, indicating that the system is converging toward a 1:1 HS:LS ratio where the moment would be 1.91 cm^3^ K mol^–1^. On further cooling χ_M_*T* decreases abruptly to 1.03 cm^3^ K mol^–1^, with the transition temperature *T*_1/2,LT_↓= 83 K (low-temperature (LT) phase), indicating a full conversion to the *S* = 1 (LS) state. Below 25 K, the χ_M_*T* value falls to 0.28 cm^3^ K mol^–1^, which is attributed to zero-field splitting of the ^3^T_1_ ground state. The two-step transition is fully reversible over multiple thermal cycles in single-crystal or polycrystalline samples with hysteresis at both steps. Values of *T*_1/2,LT_↑= 86 K and *T*_1/2,INT1_↑= 140 K are recorded on warming with hysteresis widths of 3 K, centered at 84.5 K, and 1 K, centered at 139.5 K, respectively. At 350 K, the initial χ_M_*T* value of 2.60 cm^3^ K mol^–1^ is recovered.

We use the non-symmetry-breaking order parameter , which is convenient for monitoring the stepwise spin state conversion.^52,53,80^ In the HT and INT2 phases molecules are mainly HS and *q*_spin_ ≃ 1. In the INT1 phases, *q*_spin_ ≃ 0 due to the 1:1 HS:LS ratio. In the LT phase *q*_spin_ ≃ −1, as the molecules are mainly LS. As was recently explained,^52^ stepwise SCO may be due to antiferroelastic coupling between inequivalent spin-active sites or to symmetry breaking, where spin-active sites are spatially ordered on the step. To understand the process coming into play, it is therefore mandatory to perform a detailed structural analysis, as discussed hereafter.

The thermal dependence of the derivative of the temperature-dependent molar magnetic susceptibility, d*χ*_M_*T/*d*T*, the molar magnetic susceptibility, χ_M_, and the inverse molar magnetic susceptibility, χ_M_^–1^, also confirm this stepwise behavior, as shown in Figures S1 and S2. Thus, we have identified two first-order transitions: one is centered at 84.5 K with a thermal hysteresis window of 3 K, and the second is centered at 139.5 K with a thermal hysteresis window of 1 K. The thermal hysteresis windows are of width comparable to those previously observed in other Mn^3+^ SCO complexes.^29,30,76,81^ However, this is the first example of two-step hysteretic SCO behavior in a Mn^3+^ SCO complex. Additionally, we note that the hysteresis loops have an asymmetric shape, which is also characteristic of coupling between symmetry breaking and SCO.^53^

In three of the four phases (HT, INT2 and INT1), four Mn^3+^ sites are present in the unit cell, while in the LT phase, two Mn^3+^ sites are present. In the INT1 phase, two Mn^3+^ are in the HS *S* = 2 and two are in the LS *S* = 1 state. In the LT phase, the two Mn^3+^ sites are in the LS *S* = 1 state (*vide infra*). The contents of the unit cell of each phase and the respective spin states of each independent Mn^3+^ site are represented by colored ellipses in Figure 1. The larger red and yellow ellipses represent non-symmetry-related HS *S* = 2 Mn^3+^ sites. The smaller blue and green spheres represent LS *S* = 1 Mn^3+^ sites.

### Thermochromism

**Figure 2:**
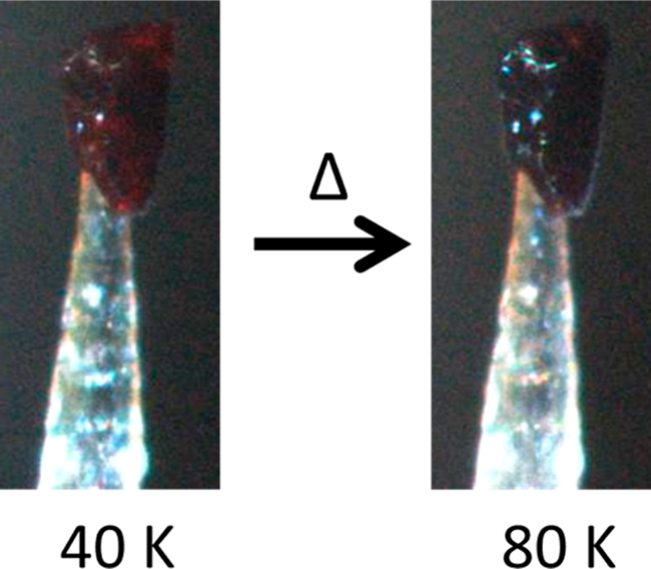
Color change of a single crystal of complex **1** mounted on a cactus needle used for the single-crystal X-ray diffraction experiment at 40 K (left, translucent ruby red) and 80 K (right, dark red) in a helium gas stream.

Complex **1** undergoes a weak reversible color change from translucent dark ruby red to dark red when it is warmed from 40 K (LT phase, LS state) to 80 K (border of LT/INT1 phase) (Figure 2). Phase coexistence between the LT and the INT1 phases may be present, influencing the overall electronic configuration and hence the color of the crystal. The weak color change was observed when the single crystal was mounted on a cactus needle used for the single-crystal X-ray diffraction experiment. It was only observed when the crystal was at temperatures far from the lower hysteresis window and when the crystal was mounted directly into a cold helium gas stream. With liquid nitrogen, the lowest temperature that could be reliably achieved was 82 K, and no apparent visible color change was observed. It is very rare to observe color changes in Schiff base Mn^3+^ SCO complexes, and a recent study on a similar Mn^3+^ complex described a much more pronounced color change of translucent red to dark red between the fully *S* = 1 and *S* = 2 states.^82^

### Structural Studies

**Figure 3:**
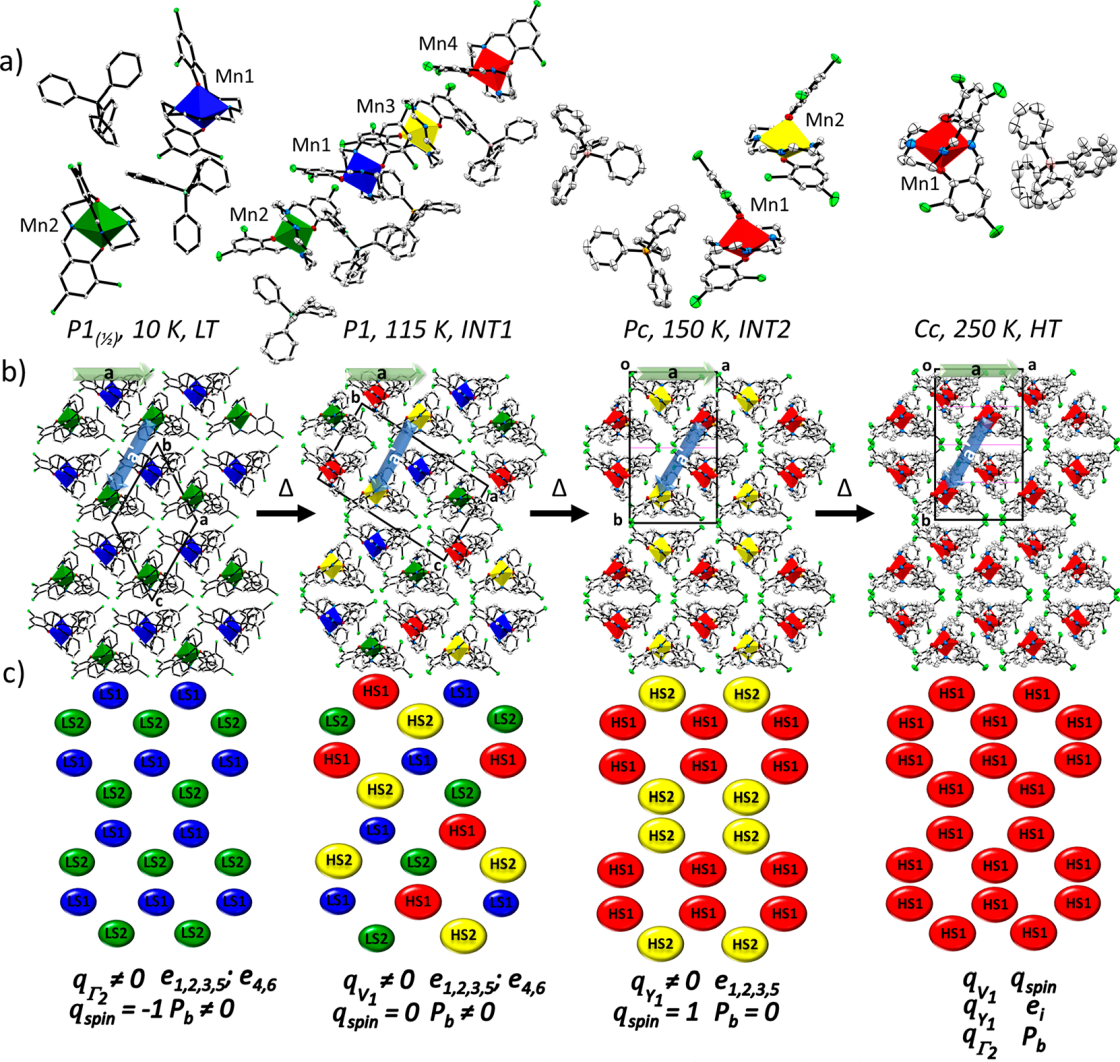
Perspective view of the LT *P*1_(1/2)_ (10 K), INT1 *P*1 (115 K), INT2 *Pc* (150 K), and HT *Cc* (250 K) structures of complex **1** and emergent order parameters (*q*) and polarization (*P*). (a) View of the asymmetric unit with distorted [MnN_4_O_2_]^+^ units shown as polyhedra with the following color coding: HS1 (red), HS2 (yellow), LS1 (blue), and LS2 (green). (b) The same structures shown in a layered crystal packing arrangement and also the relationships between the conventional unit cells of each structure. Atoms are shown at 50% atomic probability distributions for ellipsoids. Hydrogen atoms are omitted for clarity. (c) Simplified representation of the four phases in terms of the Mn^3+^ atoms alone, with the same color coding as in (a). The symmetry-breaking events between the *Cc* phases and each of the other phases are represented with their respective thermodynamic order parameters, *q*. The *Pc, P*1 and *P*1_(1/2)_ structures are all related by a group–subgroup relationship to the same parent *Cc* cell. For *Cc* → *Pc*, the phase transition is purely structural in character, *q*_Y_1__, and no change in spin state order parameter, *q*_spin_, is observed. By symmetry, the global polarization in the *Cc* and *Pc* space groups lies in the (*a*,*c*) plane, with the net polarization vector along *b, P*_b_, equal to zero. The *Cc* → *P*1 transition involves coupling between the structural order parameter, *q*_V_1__, and the spin state order parameter, *q*_spin_. The SCO event involves changes in spin state of two of the four Mn^3+^ cations in the asymmetric unit. The *Cc* → *P*1_(1/2)_ phase transition involves a structural order parameter, *q*_Γ_1__, and coupling to a change in the spin state order parameter, *q*_spin_. This is different from the spin state changes observed in the *Cc* → *P*1 phase transition, since all of the Mn^3+^ complex cations in the asymmetric unit undergo the HS state → LS state conversion. In each case, *e*_*i*_ represents components of the spontaneous strain tensor that are not zero (see section S3 in the Supporting Information).

Single-crystal X-ray diffraction data were collected at various temperatures within each of the four regions outlined on the χ_M_*T* versus *T* plot in Figure 1. The results of full refinement of the structure in the stability field of the HT phase at 250 K are given in Table S3. The structure was refined in the noncentrosymmetric polar monoclinic space group *Cc*, and the asymmetric unit comprised one independent mononuclear [Mn^III^L1]^+^ complex cation and one disordered tetraphenylborate BPh_4_^–^ counteranion (Figure S3). The coordination sphere around the Mn^3+^ ions in the HT phase is analogous to that of the previously reported complexes. The geometry can best be described in terms of a compressed distorted octahedron formed by the *trans* anionic phenolate oxygen donors and pairs of *cis*-amine and *cis*-imine nitrogen atoms in the equatorial plane. The Mn–N_imine_, Mn–N_amine_, and Mn–O_phen_ bond lengths measured at 250 K are summarized in Table S4. As we only observed an elongation of the bond lengths in the Mn–N equatorial plane and Mn–O_phen_ showed no sign of elongation, it is fair to assume that one electron occupies the d_*x*^2^–*y*^2^_ antibonding orbital. The bond lengths observed are in the range expected for HS *S* = 2 Mn–N bonds,^75−77,79,83^ and the spin state is in line with the observed χ_M_*T* value measured at 250 K. Figure S14 shows variable-temperature single-crystal X-ray diffraction data for the unit cell parameters measured from 250 to 83 K in cooling mode and 83 to 200 K in warming mode in 3 K intervals. There are obvious but continuous changes in the trend of unit cell parameters at ∼204 K, associated with a symmetry breaking from *Cc* to *Pc*, characterized by the appearance of *hkl* (and *h*0*l* reflections) with *h* + *k* odd below 204 K (Figure S15). The *Cc* phase above 204 K is a high-spin high-symmetry phase (HS) with four symmetry-equivalent HS molecules in the unit cell (Figure 3).

We performed a full data collection at 150 K, i.e. in the stability field of the INT2 phase, on the same single crystal as that measured at 250 K. The structure was refined in the noncentrosymmetric polar monoclinic space group *Pc*. Due to the reduction in symmetry, the asymmetric unit comprises two independent mononuclear [Mn^III^L]^+^ complex cations and two tetraphenylborate, BPh_4_^–^, counteranions (Figure 3a and Figure S4). A slight increase in bond lengths was observed, especially for Mn2–N_imine_ and Mn2–N_amine_, in comparison with the equivalent bond lengths observed at 250 K (Table S4). Both Mn^3+^ complex cations are in the HS *S* = 2 state. This may account for the slight increase in χ_M_*T* at 150 K in comparison with 350 K, with a weak change in slope around 205 K (Figure 1). This *Pc* phase corresponds to a HS low-symmetry phase (INT2). There are four HS molecules within the unit cell, splitting into two symmetry-equivalent HS1 and two symmetry-equivalent HS2 sites due to the *c* glide plane.

Lattice parameter variations show a steep decrease in *a*, increases in *b* and *c*, and a steep increase in the β angle below the transition at ∼140 K (Figure S14). The diffraction patterns show *h*0*l* reflections with *l* odd appearing in the *h*0*l* plane, which is characteristic of the loss of the *c* glide plane below 140 K (Figure S15) and, therefore, of a symmetry reduction toward a *P*1 phase. This INT1 phase has different symmetry from those of the higher temperature phases INT2 and HT. A full data set was also collected at 115 K (INT1 phase) on the same crystal. The structure was refined in the polar and chiral triclinic space group *P*1. Due to the loss of the *c* glide plane, the unit cell comprises four independent mononuclear [Mn^III^L]^+^ complex cations and four BPh_4_^–^ counteranions (Figure 3a and Figure S5). Two of the four Mn^3+^ complex cations are in the LS *S* = 1 state, and two are in the HS *S* = 2 state (Table S4). The donor atom bond lengths clearly show that, at 115 K, a significant decrease occurred in two of the four sets of Mn–N_imine_ and Mn–N_amine_ bond lengths, while the average Mn–O_phen_ bond length was more or less unchanged. This correlates well with the intermediate value of χ_M_*T* that is observed below 140 K. The *P*1 (INT1) structure has four independent molecular sites: two mainly HS and two mainly LS. This phenomenon results from the coupling terms between the SCO and the symmetry change.^41,52,80^

A full data set was collected at the lowest temperature we could reliably access with liquid nitrogen, 82 K (LT phase), using the same crystal as for the 115 K measurement. An analysis revealed the disappearance of some Bragg peaks at lower temperature in comparison to the structure measured at 115 K. The structure was refined in the triclinic polar and chiral space group *P*1 but with a unit cell that is only half the volume of the *P*1 structure measured at 115 K. The difference is due to a halving of the *c* parameter, and we denote the LT phase as *P*1_(1/2)_. The unit cell comprises two independent mononuclear [Mn^III^L]^+^ complex cations and two BPh_4_^–^ counteranions (Figures 3a and Figures S6 and S7). Both Mn^3+^ complex cations are in the LS *S* = 1 state (Table S4). A significant overall decrease in the Mn–N_imine_ and Mn–N_amine_ bond lengths was observed in comparison to the average for the HT, INT2, and INT1 phases. The χ_M_*T* value of around 1.03 cm^3^ K mol^–1^ below 83 K confirms this observation. Data collection was conducted on a single crystal of complex **1** at 10 K using helium for cooling to confirm the LT phase observed at 82 K (Table S3). An investigation of diffraction patterns from both the LT *P*1_(1/2)_ and INT1 *P*1 phases revealed the presence of twinning, which develops as a consequence of the ferroelastic transition from monoclinic to triclinic. Therefore, during this transition ferroelastic twin walls, i.e. regions between crystallographic twins accompanied by domain formation, develop.

Bond length variations for Mn–N donors between HS *S* = 2 and LS *S* = 1 states are ∼0.13 Å, which is typical for Schiff base N_4_O_2_^2–^ Mn^3+^ SCO complexes.^29,75−78^ SCO in these types of Mn^3+^ Schiff base complexes is manifested as a change in trigonal distortion angle, Φ, and octahedral distortion parameter, Σ. Both parameters are used to estimate the magnitude of the deformation of the coordination geometry with respect to a perfect octahedron with Σ, Φ = 0. The larger the values for Σ and Φ, the weaker the ligand field, which will stabilize the HS *S* = 2 state for the Mn^3+^ ion. The parameters that characterize the extent of the structural changes induced by the SCO behavior are summarized in Table 1. The spin state of the [Mn^III^L]^+^ complex cation in each case is also indicated. Only weak hydrogen-bonding interactions between the Mn^3+^ complex cation(s) and the BPh_4_^–^ counteranion(s) were found in the HT (250 K), INT2 (150 K), INT1 (115 K), and LT (10 K) phases (Figures S8–S12). A full Hirshfeld surface analysis, mapped over *d*_norm_, of complex **1** shows that the three main contributions to the intermolecular interactions are H···H, H···Cl, and H···C, with a decrease in H···Cl and an increase in H···H interactions in the LT phase in comparison to the INT and HT phases (Figure S13). The slight changes in intermolecular interactions may generate a change in lattice pressure that seems to directly affect the spin state and may explain the hysteretic behavior of complex **1**.

**Table 1:** Calculated Distortion Parameters and List of Spin States for Each Asymmetric [MnL]^+^ Cation in Complex **1** at 10, 82, 115, 150, and 250 K

		temperature				
param	Mn site	10 K	82 K	115 K	150 K	250 K
space group		*P*1(1/2)	*P*1_(1/2)_	*P*1	*Pc*	*Cc*
*Z*′^a^		2	2	4	2	1
						
ΣMn (deg)^b^	Mn1	36.76	37.76	43.92	69.71	63.95
	Mn2	39.66	39.29	53.35	63.28	
	Mn3			67		
	Mn4			74.6		
						
Φ (deg)^c^	Mn1	120.76	126.42	143.23	259.87	240.53
	Mn2	120.76	122.12	180.54	237.27	
	Mn3			253.83		
	Mn4			271.92		
						
ζ (Å)^d^	Mn1	0.395	0.422	0.481	0.783	0.774
	Mn2	0.408	0.413	0.532	0.769	
	Mn3			0.77		
	Mn4			0.787		
						
spin state	Mn1	*S* = 1	*S* = 1	*S* = 1	*S* = 2	*S* = 2
	Mn2	*S* = 1	*S* = 1	*S* = 1	*S* = 2	
	Mn3			*S* = 2		
	Mn4			*S* = 2		

a *Z*′ is the number of independent sites in the asymmetric unit.

b ΣMn is the sum of the deviation from 90° of the 12 *cis* angles of the MnN_4_O_2_ octahedron.

c Φ is the sum of the deviation from 60° of the 24 trigonal angles of the projection of the MnN_4_O_2_ octahedron onto the trigonal faces.

d ζ is the distance distortion parameter, which is the sum of the deviation from individual M–X bond distances with respect to the mean metal–ligand bond distance.^84^

Different representations of the striped (spin state) order of the Mn^3+^ complex cations in each of the four phases are shown as packing diagrams viewed along the *c* axis for the HT *Cc* phase, with the other phases oriented accordingly, in Figure 3b and as a graphical representation of the Mn^3+^ SCO centers in Figure 3c.

### Group–Subgroup Analysis, Order Parameters, Tensors, and Formal Strain Analysis

It is well understood that changes in the elastic constants of single crystals at phase transitions depend primarily on the form and strength of coupling between the driving order parameters and strain.^85^ In addition to the well-known coupling of a symmetry-breaking order, the non-symmetry-breaking order parameter *q*_spin_ also contributes to the volume strain. This is well illustrated by the large bond contractions and consequently lattice strains exhibited by many materials undergoing SCO without a symmetry change.^52−54^ In a recent paper by Shatruk et al., structural phase transitions coupled to SCO have been reviewed.^48^ Three classifications were made where various degrees of coupling between the SCO behavior and the structural phase transition were defined. In our case, the structural phase transition sequence is most likely due to changes in Jahn–Teller distortions on adjacent Mn^3+^ cationic molecules, which in turn induce a strain. Collet et al. also discussed the role of the elastic coupling between SCO and symmetry-breaking order parameter.^52,53^ Biquadratic coupling stabilizes the intermediate HS–LS ordered phase, while linear-quadratic terms may stabilize an average HS fraction different from 1/2 on the step. Because of the strong coupling between the SCO and structural phase transition, the two SCO steps occur in an abrupt manner in complex **1**.

The transitions can be analyzed from the perspective of Landau theory, for which the relevant order parameters and coupling with strain are described in the Supporting Information. Phase transitions of a crystal in which there is a group–subgroup relationship for the change in symmetry display coupling between a thermodynamic order parameter, *q*_struct_, and strain components, *e*_i_,^86^ (in Voigt notation).

**Figure 4:**
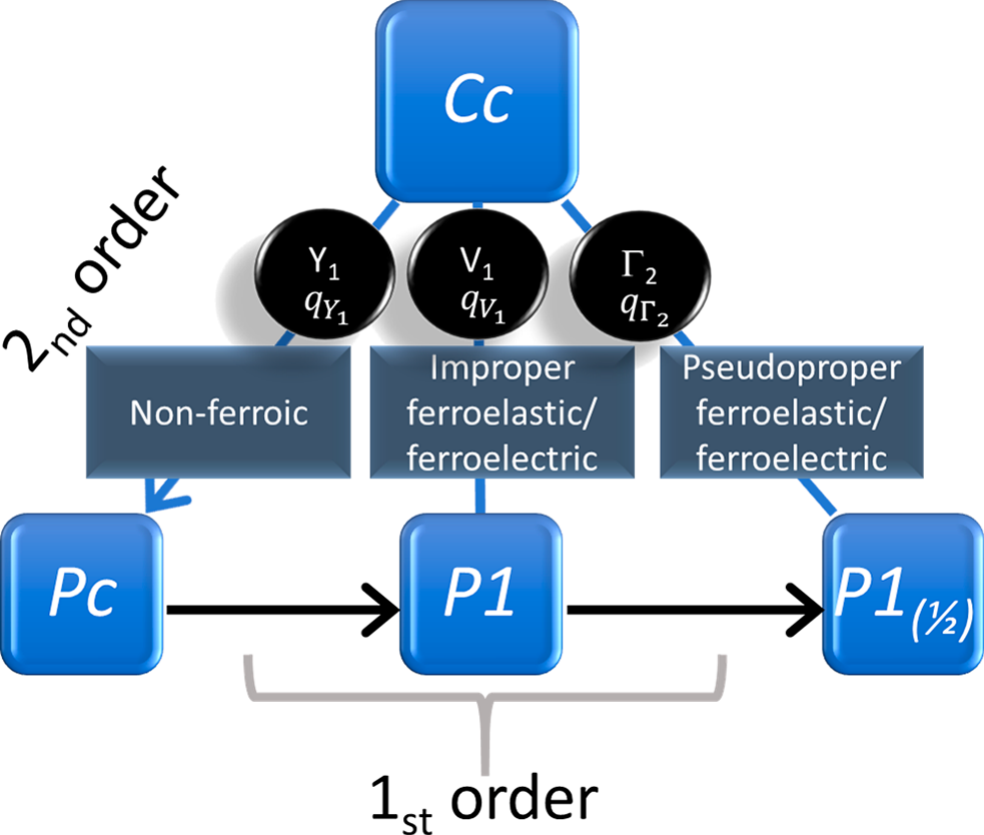
Schematic representation of group–subgroup relationships between the four phases of [Mn^III^(3,5-diCl-sal_2_(323))]BPh_4_ (**1**). *Pc*, *P*1, and *P*1_(1/2)_ are each subgroups of *Cc* (blue lines) but do not have a group–subgroup relationship between them. Arrows on solid lines indicate the sequence of transitions with decreasing temperature. The *Cc* and *Pc* structures are related by the order parameter *q*_Y_1__, which belongs to the irrep Y_1_, and the transition is allowed by symmetry to be second order. The *Cc* and *P*1 structures are related by the order parameter *q*_V_1__, which belongs to the irrep V_1_. *Cc* and *P*1_(1/2)_ structures are related by the order parameter *q*_Γ_2__, which belongs to the irrep Γ_2_. In the absence of group–subgroup relationships between the three lower temperature structures, transitions between them must necessarily be first order in character.

The parent structure for the sequence of phase transitions in complex **1** is *Cc* and, as summarized in Figure 4, each of *Pc* (INT2), *P*1 (INT1) and *P*1_(1/2)_ (LT) are subgroups of *Cc*. The *Cc* (HT) → *Pc* (INT2) transition is allowed by symmetry to be second order in character, but a group–subgroup relationship does not exist between Pc (*INT2*) and *P*1 (INT1) or between *P*1 (INT1) and *P*1_(1/2)_ (LT) so that the *Pc* (INT2) → *P*1 (INT1) and *P*1 (INT1) → *P*1_(1/2)_ (LT) transitions are necessarily first order (reconstructive). For the *Cc* → *Pc* transition we specify the thermodynamic order parameter as *q*_Y_1__, the amplitude of which measures deviations from *C*-face centering due to a structural instability involving displacements of molecules HS1, HS2, and counteranions. Lowest order strain coupling terms have the form λ_*i*_*e*_*i*_*q*_Y_1__^2^ for *i* = 1, 2, 3, 5 and λ_*i*_*e*_*i*_^*2*^*q*_Y_1__^2^ for *i* = 4, 6, where *λ*_*i*_ are coupling coefficients (see Section S3 in the Supporting Information). Coupling of the form λ_*i*_*e*_*i*_*q*_Y_1__^2^ gives *e*_*i*_ ∝ *q*_Y_1__^2^ in simplest form.^86^ This transition does not give rise to a symmetry-breaking shear strain or to electric/magnetic polarization and is therefore classified as coelastic and nonferroic.

For the *Cc* → *P*1 transition we specify the thermodynamic order parameter as *q*_V_1__, and the lowest order terms for coupling with strains are λ_*i*_*e*_*i*_*q*_V_1__^2^, *i* = 1–6. This would be an improper ferroelastic, improper ferroelectric transition, since the symmetry-breaking shear strains, *e*_4_ and *e*_6_, and polarization, *P*, each couple with *q*_V_1__^2^ rather than with *q*_V_1__. For the *Cc* → *P*1_(1/2)_ transition we specify the thermodynamic order parameter as *q*_Γ_2__, and the lowest order strain coupling terms are λ_*i*_*e*_*i*_*q*_Γ_2__^2^, *i* = 1, 2, 3, 5 and λ_*i*_*e*_*i*_*q*_Γ_2__, *i* = 4, 6. This transition would be ferroelectric and pseudoproper ferroelastic, where the term pseudoproper refers to the fact that the symmetry of the symmetry-breaking strains is the same as the symmetry of the structural order parameter that drives the transition.

**Figure 5:**
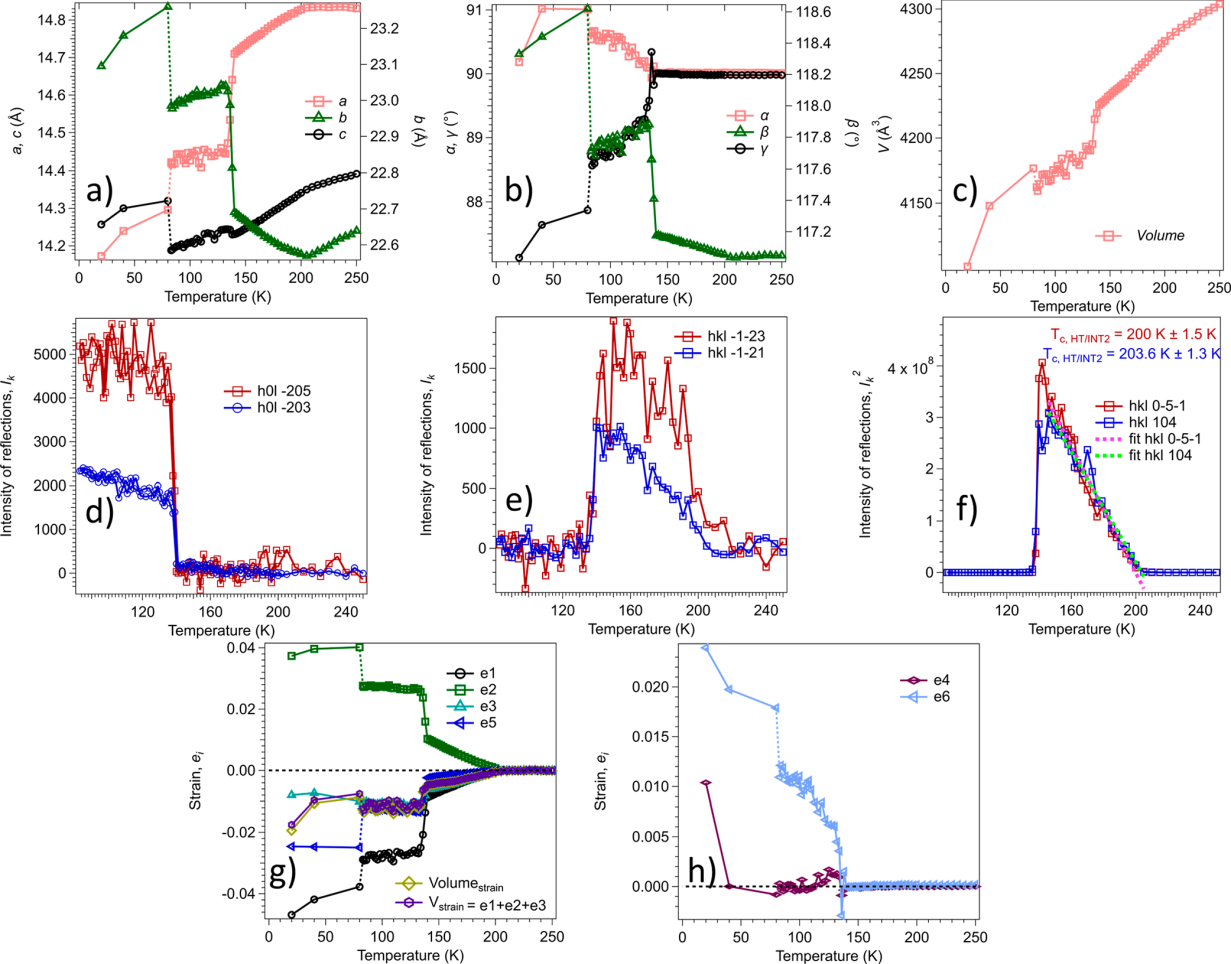
Changes in unit cell parameters (a) *a*, *b*, and *c*, (b) α, β, and γ, and (c) volume, from single-crystal measurements of complex **1** between 250 and 20 K. (d) Intensity of superstructure reflections appearing at the first-order *Pc* (INT2) → *P*1 (INT1) transition. The Bragg peaks were indexed in the *P*1_(1/2)_ space group setting. Intensities of superstructure reflections through the *Cc* (HT) → *Pc* (INT2) transition, corresponding to Bragg peaks (*hkl*) with *h* + *k* odd shown for (e) the weaker *hkl* = −1–23 and −1–21 reflections and (f) the stronger *hkl* = 0–5–1 and 104 reflections, which appears in diffraction patterns from the *Pc* phase. The Bragg peaks were indexed in the *Cc* space group setting. The data show *I*_k_^2^ ∝ (*T*_c_ – *T*), within experimental uncertainty, and *T*_c_ = 202 ± 3 K. (g) Temperature dependence of strain components for the *Pc* and *P*1 structures, as defined with respect to the parent *Cc* structure. All of the strains vary continuously through the *Cc* (HT) → *Pc* (INT2) transition at ∼204 K, discontinuously through the Pc (INT2) → *P*1 (INT1) transition at ∼135 K, and discontinuously through the *P*1 (INT1) → *P*1_(1/2)_ (LT) transition at ∼80 K.

Lattice parameters determined by single crystal X-ray diffraction show a small increase in *a* and decrease in *b* and *c* with decreasing temperature. At ∼204 K there is a change in slope for all the parameters, indicating a change in structure (Figure 5a–c). The change in the Bragg peaks *hkl* associated with the appearance of superlattice reflections on cooling is shown in Figure 5d,e. In particular the development of superlattice reflections below ∼204 K (*T*_c,HT/INT2_) associated with the *Cc* (HT) → *Pc* (INT2) transition, corresponding to Bragg peaks *hkl* with *h* + *k* odd, is shown in Figure 5e,f. The square of the intensities of these scales linearly with temperature, and hence, the thermodynamic character of the transition is best described as Landau tricritical (*I*_k_^2^ ∝ *q*_Y_1__^4^ ∝ (*T*_c_ – *T*)) with *T*_c,HT/INT2_ = 202 ± 3 K. For this analysis the best reflections from the *hkl* family were taken into account.

A continuous decrease in *a* and *c* and an increase in *b* were observed down to ∼140 K, where a steep decrease in *a* and increases in *b* and *c* were observed. The appearance of superstructure reflections below 140 K is evident from Figure S15. The lower limit for the temperature that can be reached with the nitrogen cryostat in this experiment is 82 K. A helium cryostat was used for measuring the unit cell parameters at 80, 40, and 20 K on a different single crystal, hence the dashed lines in the plot below 82 K. Measurements from the first single crystal between 240 and 83 K cannot necessarily be related with high precision to the unit cell of the second single crystal measured at 80, 40, and 20 K, but the discontinuities are consistent with first-order transitions at ∼140 and ∼80 K (Figure 5a,b).

A change in spin state by itself does not necessarily result in any change in symmetry; thus, the simplest spin order parameter, *q*_spin_, transforms as the identity representation. As it is the high-spin, high-symmetry phase that is taken as a reference, the convenient definition of *q*_spin_ given above corresponds to *q*_spin_ = 2γ – 1, where γ is the fraction of the high-spin state, varying between 0 and 1, which we use to specify the value of this order parameter.^52,53,80^ If the spin state transition is linked to a symmetry-breaking phase transition with order parameter *q*_struct_ and coupling with strain *e*_*i*_, the overall free energy change can be expressed in terms of a Landau type expansion in generalized form as
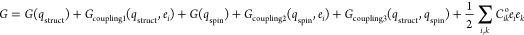
1

Here, the last term describes the Hooke’s law elastic energy; *C*_*ik*_^o^ represents the elastic constants of the reference structural state (*Cc* in this case) and is not considered any further. Three different coupling terms are specified to draw attention to the fact that the two order parameters couple separately with strain as well as with each other.

Writing the full potential is complex in the present case, as there are various competing false ground states associated with SCO and/or different structural instabilities. The generalized expression nevertheless explains the overall pattern of behavior. On this basis, *q*_struct_ may correspond to *q*_Y_1__, *q*_V_1__, or *q*_Γ_2__, coupling between SCO, and symmetry breaking and *G*_coupling3_(*q*_struct_, *q*_spin_), may include several terms, as explained in the framework of coupled SCO and symmetry breaking.^52^ The linear-quadratic coupling term *q*_spin_*q*_Y_1__^2^ in the case of the tricritical HT → INT2 phase transition affects the SCO only weakly, as a slight change in slope in the thermal spin state conversion near 205 K (Figure 1), driven by the evolution of *q*_Y_1__. On the other hand, the term *q*_spin_*q*_Γ_2__^2^ is responsible for the discontinuous evolution of the high-spin fraction from INT1 to LT because *q*_Γ_2__ emerges discontinuously at the reconstructive transition. Biquadratic coupling terms such as  play an important role in stepwise transitions driven by symmetry breaking, as they stabilize half-conversion around *q*_spin_ = 0.^52,80^ The symmetry-breaking order parameter *q*_V_1__ is therefore associated with the appearance of spin-state concentration waves^41,50^ that form in the INT1 phase. This corresponds to a long-range ordering of molecules in HS and LS states over molecular sites HS1 and LS1, which are symmetry-equivalent at higher temperature. The symmetry-breaking order parameter *q*_V_1__ scales as the difference of HS population on these sites: *q*_V_1__ ∝ |*q*_spin, HS1_ – *q*_spin, LS1_|. On this step the linear-quadratic coupling term *q*_spin_*q*_V_1__^2^ slightly shifts the equilibrium *q*_spin_ from 0. Various types of spin-state concentration waves, forming various patterns, were theoretically found by Cruddas et al. in the framework of a microscopic model based on elastic interactions.^51^ However, in addition to the HS–LS pattern appearing in INT1 phase, the ferroelastic distortion between monoclinic and triclinic INT1 phases plays a key role, especially for the magnetoelectric coupling discussed below.

Spontaneous strain variations through the full sequence of transitions, as calculated from changes in lattice parameters using equations given in the Supporting Information, are shown in Figure 5g,h. Linear extrapolations were used to determine values of the reference parameters, and a simple test of the efficacy of this is provided by comparison of values for *V*_s_ obtained directly using eq S11 with values obtained using *V*_s_ = *e*_1_ + *e*_2_ + *e*_3_. As shown in Figure 5g, the two variations of *V*_s_ are the same within reasonable experimental uncertainty. The overall pattern of strain variations is consistent with a continuous transition at ∼204 K and discontinuous transitions at ∼135 and ∼80 K.

Two separate generic strain coupling terms, *G*_coupling1_ and *G*_coupling2_, have been included in eq 1 to emphasize the fact that the total variation of spontaneous strain through the full sequence of transitions includes separate contributions from coupling with each of the two order parameters, *q*_struct_ and *q*_spin_. Without additional information on the evolution of each of the different order parameters, it is not possible to separate out their different coupling contributions to the total strain, but some general points are clear. First, *q*_spin_ = 0 in the stability field of the *Pc* (INT2) structure and the individual strains vary continuously up to ∼±1% by coupling only with the structural order parameter *q*_Y_1__. Second, an initial expectation would be that increases in *q*_spin_ contribute predominantly to reductions in volume: i.e., to negative volume strains. The data for *V*_s_ in Figure 5g indeed show abrupt increases in magnitude from ∼−0.5% in the stability field of the *Pc* (HT) structure to ∼−1% and ∼−2% in the stability fields of the *P*1 (INT1) and *P*1_(1/2)_ (LT) structures, respectively, which both have *q*_spin_ ≠ 0. More or less constant values of *V*_s_ through the full temperature intervals of the two triclinic structures imply that the proportion of sites at which Mn is in the high-spin state and the proportion of sites at which Mn is the low-spin state remain approximately constant in each of them. Finally, the dominant ferroelastic shear strain below ∼140 K is *e*_6_, which increases up to 1% in the stability field of the *P*1 (INT1) structure and up to ∼2% in the stability field of the *P*1_(1/2)_ (LT) structure; *e*_4_ remains close to zero at the lowest measurement temperature (Figure 5h).

The three coupling terms in eq 1 are also important in relation to the structure and properties of ferroelastic domain walls in the *P*1 (INT1) and *P*1_(1/2)_ (LT) structures, since these represent narrow regions across which there are steep gradients in strain. There is a total strain contrast of |2*e*_6_| from one domain to the next across each wall and, in principle, *q*_V_1__ or *q*_Γ_2__ as *q*_struct_ and *e*_6_ as the symmetry-breaking strain will be zero at its center. Since *q*_struct_ and *q*_spin_ are coupled, there will also be gradients in the degree of spin ordering within the walls, depending on the strength of the term *G*_coupling3_ for each of *q*_V_1__ and *q*_Γ_2__. It follows that any magnetic, ferroelectric, or magnetoelectric properties of the material which depend on the ordered arrangement of atoms with high and low spins will be locally different within the domain walls in comparison with the properties of bulk crystals. It follows also that the density within the domain walls might be as much as 2% lower than that within individual domains if *q*_spin_ is suppressed to zero, providing the possibility of highly localized preferred sites for impurity atoms and defects or of pathways for enhanced diffusion of dopant atoms, for example. In this context it is worth noting that the strain contrast across ferroelastic domain walls in the *P*1_(1/2)_ (LT) structure will be double that across domain walls in the *P*1 (INT1) structure and hence that contrasts in other properties will also be enhanced.

### Ferroelastic Behavior

The RUS technique has previously been used to follow variations in elastic properties of materials that exhibit spin crossover behavior with and without symmetry-breaking phase transitions.^30,54,55,87^ Values of elastic moduli scale with *f*^2^, where *f* represents the frequency of an individual peak in spectra which record predominantly shear mode acoustic resonances of samples with dimensions in the vicinity of ∼1 mm. Acoustic loss is expressed in terms of the inverse mechanical quality factor, *Q*^–1^, which depends on the widths at half-height of the resonance peaks.

**Figure 6:**
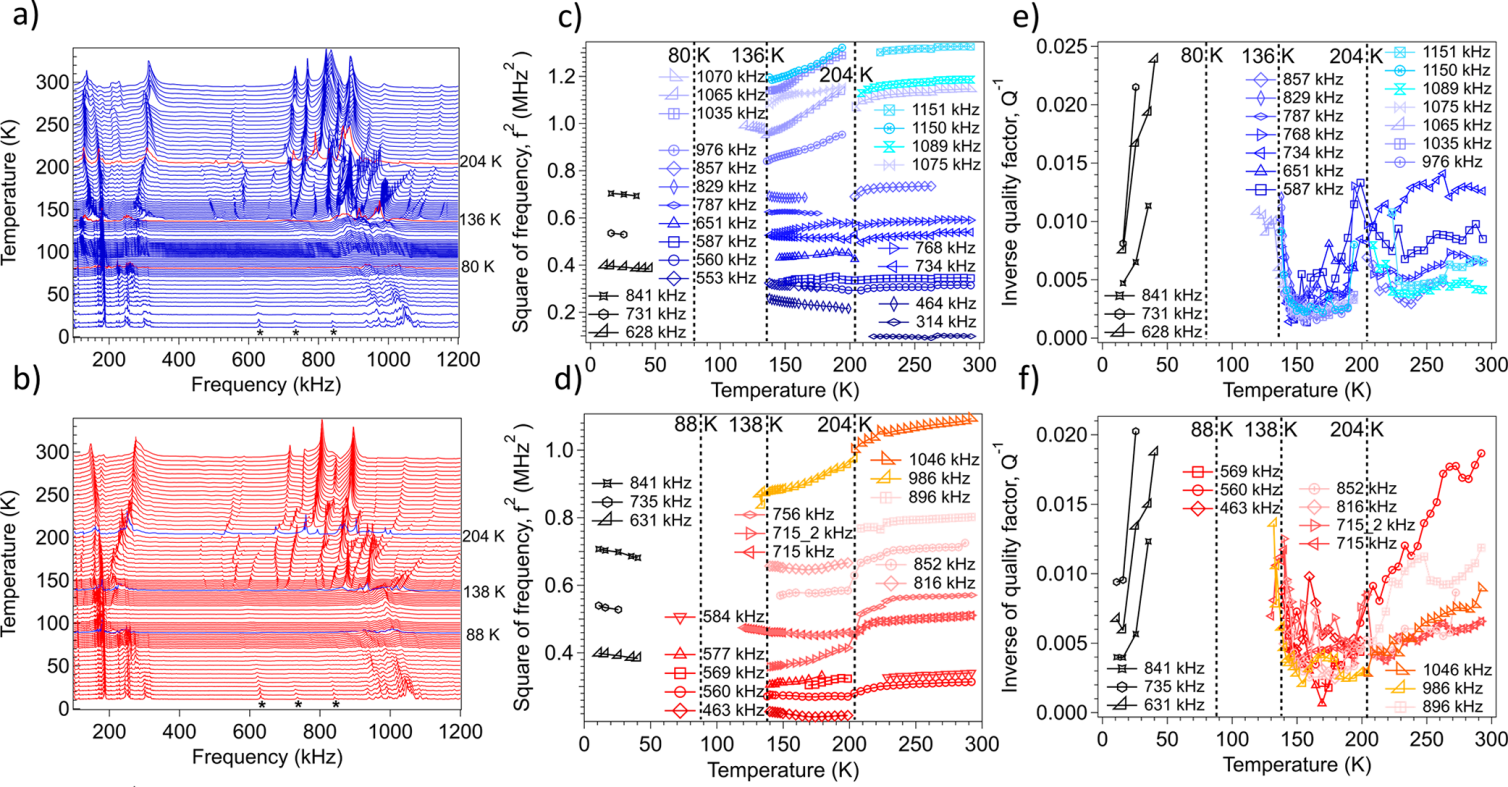
(a, b) RUS spectra as a function of frequency for a single crystal of complex **1**, stacked up the *y* axis in proportion to the temperature at which they were collected. The *y* axis is amplitude (volts) but has been relabeled as temperature to reveal the overall pattern of stiffening and softening. Spectra were collected during (a) cooling and (b) heating between ∼292 and ∼10 K. Highlighted spectra in both stacks indicate the expected positions of HT → INT2, INT2 → INT1 and INT1 → LT transitions, with thermal hysteresis between the cooling and warming data for the two lower transition points. Stars mark the three peaks that most clearly show the reappearance of resonances with relatively low attenuation at the lowest temperatures. (c, d) *f*^2^ and (e, f) *Q*^–1^ data from fitting of selected resonances with an asymmetric Lorentzian function. Vertical broken lines mark the continuous structural phase transition at ∼204 K and discontinuous transitions at ∼136/138 and ∼80/88 K. Data shown in black are for the three resonance peaks marked with stars in (a) and (b). Listed in the captions are approximate frequencies for each resonance peak used in the fitting procedure to obtain values of *f*^2^ and *Q*^–1^.

Two stacks of spectra collected during cooling and warming of a single crystal of complex **1** are shown in Figure 6a,b, respectively. Each spectrum has been offset up the *y* axis in proportion to the temperature at which it was collected in order to reveal the overall pattern of elastic softening/stiffening through the full transition sequence. Variations in *f*^2^ and *Q*^–^^1^ from peak fitting are shown in Figure 6c,e (cooling) and Figure 6d,f (heating). Individual resonance peaks display a small shift to lower frequencies (elastic softening) during cooling from 295 to ∼204 K, where the trend of softening generally continues. Almost all the resonance peaks disappeared abruptly at ∼136 K and then reappeared gradually below ∼80 K. When the temperature was increased back to room temperature from 7 K (Figure 6b), the same essential features were observed. Resonance peaks returned to the same positions as in the cooling sequence, confirming that the crystal survived all the transitions without cracking or undergoing other irreversible changes.

Changes in elastic constants of single crystals at phase transitions depend primarily on the form and strength of coupling between the driving order parameter with strain.^85^ The *Cc* → *Pc* transition is tricritical, and coupling of the form λ_*i*_*e*_*i*_*q*_Y_1^2^ would be expected to give rise to discontinuous softening as the crystal is cooled through the transition temperature.^82^ Instead of a discontinuity at *T*_c_ (***∼***200 K), however, individual resonances show more or less continuous decreases in frequency that is typical of situations where the order parameter does not relax on the time scale of the applied stress. Relaxation times of longer than ∼10^–6^ s (i.e. for measurements at ∼1 MHz) must derive from slow dynamics of structural changes involved in the transition mechanism. Changes in the elastic constants then vary as λ_*i*_*q*_Y_1__^2^ due to the next higher order coupling terms of the form λ_*i*_*e*_*i*_^2^*q*_Y_1__^2^. The transition is accompanied by a slight peak in *Q*^–1^ values (Figure 6e), as would be typical of acoustic loss due to critical slowing of some aspect of order parameter fluctuations as *T*_c_ is approached from above and below. A quick return to low values below the transition point occurred because ferroelastic twins do not develop at a coelastic transition.

The *Pc* → *P*1 transition at ∼140 K was marked by a sudden disappearance of most of the resonance peaks (Figure 6c,d). A few may still be weakly present in the spectra, but if so, they are notably broadened in comparison with resonance peaks from the monoclinic structures. High attenuation of this type is typical of the contribution of ferroelastic domain walls appearing below a ferroelastic transition point and remaining mobile under the influence of dynamic shear stress on a time scale of ∼10^–6^ s.^88^ Resonances from the sample became detectable again below ∼88 K, initially through their interaction with weak peaks from the sample holder. This interval of strongest attenuation was ∼80–138 K during heating and corresponds to the stability field of the *P*1 (INT1) structure, with some small hysteresis of the first-order transition points. Peaks in the primary spectra became definitely identifiable as individual resonances of the sample below ∼40 K, indicating that the domain wall mobility was reduced by pinning/freezing effects only at the lowest temperatures.

The equivalent disappearance of acoustic resonance peaks due to the mobility of ferroelastic domain walls in LaAlO_3_ under the low-stress, high-frequency conditions of an RUS experiment has been described elsewhere as “superattenuation”.^89^ On this basis, it appears that the *P*1 (INT1) structure is superattenuating while the *P*1_(1/2)_ (LT) is not. Key differences between the two structures are the proportions of Mn^3+^ in the LS state and the magnitude of the spontaneous shear strain, providing a first indication that the dynamic properties of ferroelastic domain walls in SCO structures can depend on the degree of spin ordering: i.e., on the value (in general terms) of *q*_spin_.

The mobility of ferroelastic domain walls depends on the strength of their interaction with strain fields around point defects. In the case of oxide perovskites such as LaAlO_3_, the most significant point defects are oxygen vacancies.^90,91^ A wider generalization is that thin walls will interact with point defects more strongly in comparison to thick walls^92^ so that domain wall pinning or freezing temperatures observed for different materials can be considered, in part at least, as a pointer to wall thickness. Again with LaAlO_3_ as an example, the thickness of ferroelastic domain walls has been measured by X-ray diffraction to be ∼20 Å at low temperatures^93^ and they become pinned below ∼400 K.^90,91^ No equivalent measurements have yet been made on SCO materials, but the much lower freezing temperature implied by high acoustic loss down to at least ∼35 K reported here implies a relatively weaker interaction with defects and, therefore perhaps, thicker domain walls.

### Magnetoelectric Coupling

**Figure 7:**
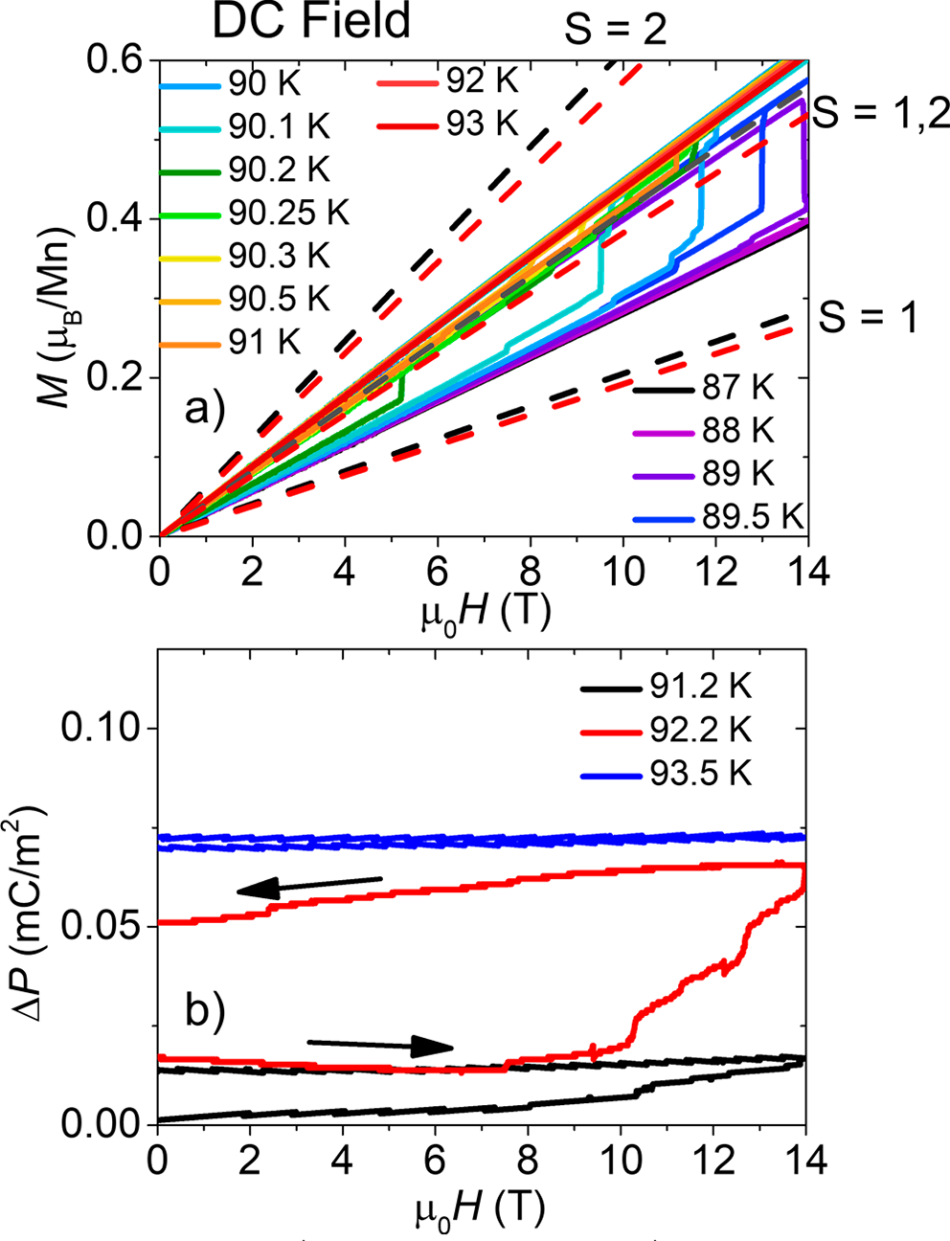
Change in (a) magnetization, *M*, and (b) electric polarization, Δ*P*, vs magnetic field, μ_0_*H*. Data were taken in magnetic fields up to 14 T in a superconducting magnet at sweep rates of 100 Oe/s for the different temperatures indicated in the legend. Arrows indicate increasing or decreasing field. Before each scan the temperature was cooled from above 100 K to the final measurement temperature. The dashed lines in (a) are calculated Brillouin functions for *S* = 1, *S* = 2, and a mixed 1:1 ratio of *S* = 1 and *S* = 2 spin states with no orbital contributions. Upper and lower lines are the lower and upper temperature limits, respectively (87 and 93 K). Data were collected using millimeter-sized single crystals of complex **1** mounted with glue to a sapphire probe with the magnetic field and electric field along the long axis. Two different crystals were measured in (a) and (b), subject to different thermal strain conditions which may have caused the difference in transition temperatures.

We have established that the SCO behavior in complex **1** can be induced by temperature. Now we investigate magnetic-field-induced SCO in **1**. The electric polarization, Δ*P* ,and magnetization, *M*, as a function of field up to 14 T are shown in Figure 7. The data in Figure 7 were taken in a superconducting magnet with slow (100 Oe/s) sweep rates. *M*(μ_0_*H*) in Figure 7a shows jumps on an increase in the field for temperatures between 88 and 90.2 K. This suggests a magnetic-field-induced SCO to a state with a higher spin value. However, a comparison of the LT data to the predicted Brillouin functions of the *S* = 1 state shows a higher magnetization value than for pure *S* = 1. After the *H*-induced transition, *M*(μ_0_*H*) follows a Brillouin function consistent with the mixed *S* = 1,2 state. A possible explanation for this discrepancy could be different amounts of strain induced by the attachment of the sample to the measurement probe.

In Figure 7b we show that the electric polarization Δ*P* also undergoes a field-induced jump with a magnetic field as low as 4 T. All of the different structural states of this material are polar and can be expected to have different electric polarizations, as was also observed in the Br analogue of this compound.^68^ A key point is that, due to the ferroelastic transition, the electric polarization, which is confined in the (*a*,*c*) plane in the monoclinic HT and INT2 phases, gets a component along the *b* axis in the INT1 and LT triclinic phases. We expect a change in electric polarization at every structural phase transition, and the temperature-dependent electric polarization is shown in Figure S17 in the Supporting Information. Figure 7 demonstrates that relatively low magnetic fields can be used to induce SCO, resulting in jumps in both magnetization and electric polarization. It is also worth noting that the magnetization and the electric polarization are hysteretic—once the spin state switches with magnetic field, it remains switched as the magnetic field is removed, provided the temperature is kept within the region of bistability of the SCO. Similar behavior was also seen in the Br analogue of this compound.^68^ This hysteresis is to be expected, given the coupling between the spin state and the symmetry-breaking order parameters.^52,53^ We note that in Figure 7a,b the temperatures at which the field-induced transitions can be observed are slightly different—this may be because different crystals were measured with different strains arising from differential thermal contraction of the sample holders. The jumps within the SCO transition in the electric polarization were also observed in the Br sample and are consistent with domain reorientations occurring during the phase transition. They are evident only in the electric polarization and not in the magnetization.

**Figure 8:**
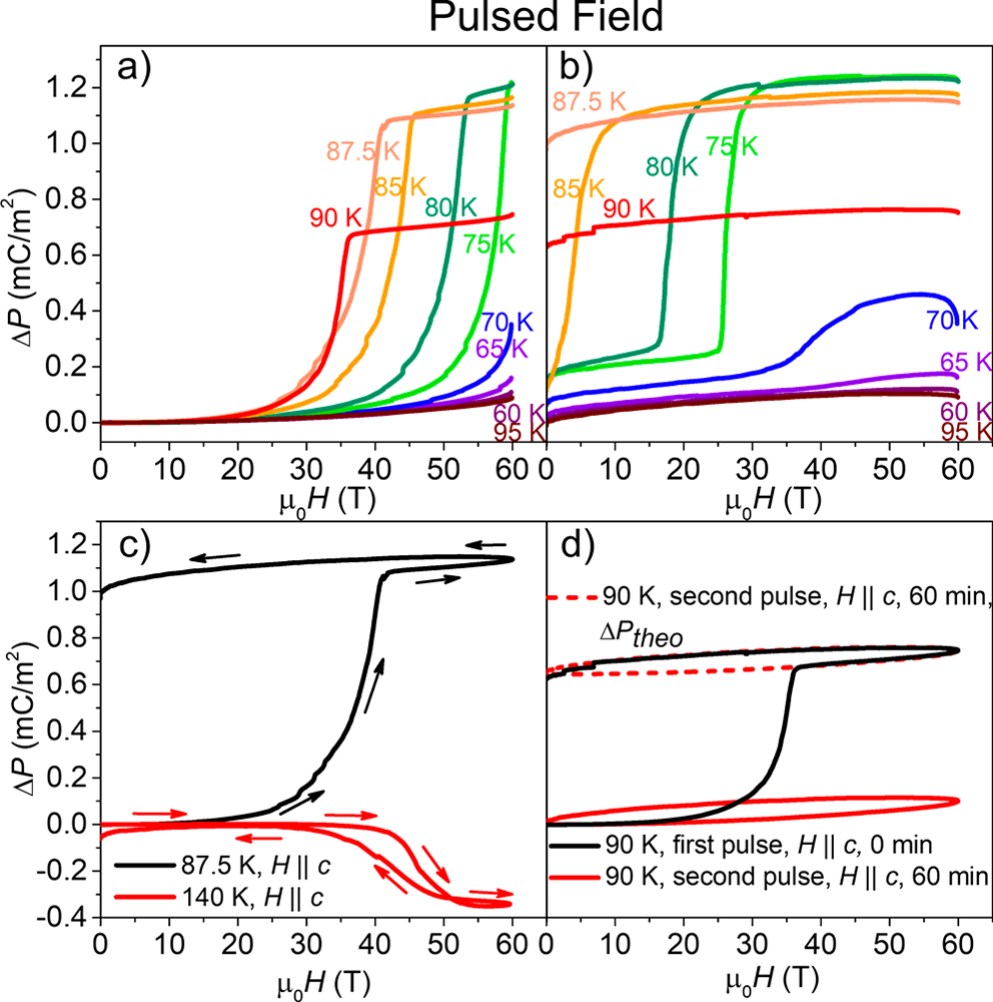
Change in electric polarization, Δ*P*, induced by a 60 T pulsed magnetic field, μ_0_*H*, applied to a single crystal of complex **1** for different temperatures as indicated. Δ*P* versus μ_0_*H* measured during (a) rising fields and (b) falling fields for temperatures between 60 and 90 K. Random errors are smaller than the line width. Systematic errors due to mechanical vibrations caused the oscillations observed in the pulsed field magnetization data. The maximum change in electric polarization induced by the 60 T pulsed magnetic field, 1.23 mC m^–2^, was observed at 75 K. (c) Change in sign of Δ*P* measured inside the two regions of bistability, the *P*1 → *Pc* (red plot) and *P*1_(1/2)_ → *P*1 (black plot) transitions. The changes in Δ*P* observed in the regions of bistability for the *P*1 → *P*1_(1/2)_ and *Pc* → *P*1 transitions were 1.14 and −0.35 mC m^–2^, respectively. (d) Δ*P* versus μ_0_*H* applied at 90 K, followed by a waiting period at 90 K for 45 min before applying a second pulse. Δ*P* reached a maximum of ∼ 0.75 mC m^–2^ during the first pulse. In the second pulse the signal showed no transition (red full line). We thus conclude that the sample remained in the HS state between pulses, and hence that the data represented by the red line should contain an additional offset (this magnetometer measures only the change in magnetization with field, not the absolute magnetization). To illustrate this, the dashed red line shows the red data with the addition of a presumed offset.

We go on to investigate the magnetic-field-induced spin crossover over a broader magnetic field range in pulsed fields. Figure 8 shows Δ*P*(μ_0_*H*) up to 60 T in millisecond pulsed fields for (Figure 8a) rising and (Figure 8b) falling fields. In these larger fields we can observe the magnetic-field-induced SCO over a wider range of temperatures from 60 to 90 K, which extends outside the bistable temperature region of the LT → INT1 SCO. Though there is hysteresis in the electric polarization between up and down sweeps of the magnetic field, it does switch back upon lowering the field outside the thermal region of bistability. However, in Figure 8c,d Δ*P*(μ_0_*H*) is shown at *T* = 87.5 and 90 K, respectively, which is within the thermal region of bistability and, thus, the switching appears to be permanent. In Figure 8d, the two magnetization pulses (black and red) are applied consecutively (with a 45 min waiting period for the magnet to cool off in between). The second pulse (red) does not show the transition, from which we conclude that the sample is still in the high-field state and has not relaxed back, consistent with being in the thermal range of bistability of the phase transition. We note that the measured quantity is the change in electric polarization Δ*P* not the absolute *P*. We conclude that the data during the second pulse should have an offset (shown as a red dotted line) to indicate that it remains in the HS state. In Figure 8c we also show Δ*P*(μ_0_*H*) at a higher temperature of 140 K, where we expect a transition from the INT2 to a field-induced state. We also see a change in electric polarization at this temperature, with a sign opposite to that of the LT → INT1 transition. Figure S18 in the Supporting Information shows the electric polarization vs temperature and magnetic field in a color contour plot for rising and falling fields.

The change in electric polarization as a function of temperature and in pulsed fields is on the order of mC m^–2^. On the other hand, in 14 T DC fields it is only 5% of that value. This may be due to only a partial transition occurring in DC fields. The origin of this magnetoelectric coupling results from the couplings between the non-symmetry-breaking spin state order parameter *q*_spin_ and the symmetry-breaking order parameter *q*_struct_. Applying a magnetic field shifts the equilibrium HS fraction (*q*_spin_), while the couplings result in a shift of the equilibrium structure (*q*_struct_) and, therefore, in a change of electric polarization.

### Comparison with [Mn^III^(3,5-diBr-sal_2_(323))]BPh_4_

Substitution of the ligand substituents provides an additional means of manipulating the properties of SCO materials. The dibromo equivalent of complex **1** has the same sequence of *Cc* (HT) → *Pc* (INT2) → *P*1 (INT1) transitions and similar properties but does not appear to have a stability field for the *P*1_(1/2)_ (LT) structure. It also does not display acoustic superattenuation in the stability field of the *P*1 (INT1) structure.^30^ The dominant effects of substituting a chloro for a bromo group are likely to be consequences simply of the change in covalent radius. The unit cell volume of the *Cc* structure at 250 K is ∼1.3% smaller in the case of the dichloro compound relative to the dibromo compound. Denser packing represented by this reduction in volume associated with reducing the size of the halo substituent presumably then hinders the reduction in volume achievable by increasing the proportion of the Mn^3+^ ions in the LS state of the *P*1_(1/2)_ (LT) structure.

A more subtle difference is that all the acoustic resonances of the dibromo compound show elastic stiffening with falling temperature (Figure 4 of ref (30)), while those of the dichloro compound show elastic softening (Figure 6). The normal expectation is that the shear elastic moduli of crystalline materials will increase as the temperature falls due to increasing density arising from the effects of normal thermal expansion. Elastic softening with falling temperature is typically an indication of proximity to some approaching structural instability. If this were related to an impending Jahn–Teller transition, for example, the softening with falling temperature would be expected to occur in only one of the elastic moduli, typical of any pseudoproper ferroelastic transition.^85^ However, the dominant trend for resonances of the dichloro crystal is softening with falling temperature, implying that most of the shear elastic moduli soften. This is more indicative of the incipient instability being related to dynamic effects in open structures such as the high-temperature polymorph of quartz ahead of the α → β transition.^85,94^ Softening with falling temperature under these circumstances is attributed to fluctuations that increase in amplitude as the transition point is approached.

The most overt difference between the two compounds, however, relates to the mobility under dynamic stress of ferroelastic domain walls. Acoustic resonances were easily identifiable in RUS spectra from the *P*1 phase of the dibromo compound.^30^ Increases in *Q*^–1^ up to ∼0.01 immediately below the *Pc* → *P*1 transition point (∼85 K) were interpreted as providing evidence of some mobility of the domain walls. However, this is significantly less attenuation than is seen in spectra from the dichloro compound, where a transition to the *P*1 structure causes the disappearance of resonances. *P*1_(1/2)_ yields values of *Q*^–1^ < 0.01 only below ∼35 K. Values of *Q*^–1^ at 10 K were found to be ∼0.001 for the dibromo compound and ∼0.005 for the dichloro compound. It is clear that the as yet undefined pinning mechanisms are sensitive to volume changes. As a speculation, it is proposed that this is a reflection of domain wall thickness—thicker in the dichloro compound than in the dibromo compound.

## Conclusions

In summary, we have reported the detection of both mobile and pinned domain walls in response to mechanical stress in two spin-state ordered thermal regimes of the Mn^3+^ Schiff base complex [Mn^III^(3,5-diCl-sal_2_(323))]BPh_4_ with a two-step hysteretic SCO. From single-crystal X-ray diffraction measurements and resonant ultrasound spectroscopy it was found that four phases exist across the 350 to 4 K temperature range. A symmetry-breaking Landau tricritical phase transition occurs from the HS *Cc* to the HS *Pc* structure and two first-order phase transitions, *Pc* → *P*1 toward an ordered HS-LS state and *P*1 → *P*1_(1/2)_ toward the LS phase. The triclinic phases are ferroelastic. Strong magnetoelectric coupling was found in the regions of bistability between the *P*1 → *Pc* and *P*1_(1/2)_ → *P*1 phases, respectively, and a reversal in sign of the electric polarization was observed between the two regions of bistability. Moreover, a memory effect for the *P*1_(1/2)_ → *P*1 transition is achieved.

These different characteristics must be correlated with the strong cooperativity (of elastic origin) observed in this compound that manifests itself in an extremely abrupt and robust spin transition. Strain coupling of spin and structural order parameters provides a likely mechanism for yielding magnetoelectric coupling in a two-step SCO complex undergoing multiple structural phase transitions and, thus, may provide a better understanding of the magnetostructural coupling that is observed in a large number of spin transition materials. Magnetic-field-induced transitions can also be observed on starting from the INT1 and LT phases.

In contrast to hard materials, the relatively soft lattices of molecular magnets have energy scales comparable to the energy scales of magnetic and electric orders, leading to a rich interplay and coupling of structural, magnetic, and electric degrees of freedom. In particular, spin state switching at spin crossovers can drive structural phase transitions in response to moderate temperatures and magnetic fields with energy scales at or below room temperature. In such systems large atomic displacements are commonly produced, creating exceptionally strong magnetoelectric coupling to the lattice and significant strain gradients and, as such, they hold great promise for the next generation of multiferroics.

## Experimental Methods

### Synthesis

[Mn^III^(3,5-diCl-sal)_2_323]BPh_4_ (complex **1**) was synthesized by addition of a solution of *N*,*N*-bis(aminopropyl)ethylenediamine (0.25 mmol, 0.0436 g) in 1/1 CH_3_CN/EtOH (15 mL) to a solution of 3,5-dichlorosalicylaldehyde (0.5 mmol, 0.0955 g) also in 1/1 CH_3_CN/EtOH (15 mL), forming a yellow solution. This solution was stirred for 15 min before a mixture of manganese(II) nitrate tetrahydrate (0.25 mmol, 0.0628 g) and sodium tetraphenylborate (0.25 mmol, 0.0856 g) in 1/1 CH_3_CN/EtOH (10 mL) was added. The resulting solution was stirred, yielding a dark brown solution of the Mn^3+^ complex resulting from air oxidation of the Mn^2+^ starting salt. Dark red block crystals of suitable quality for single-crystal X-ray diffraction were formed within 1 day upon slow evaporation of the solvent (yield: 124.0 mg, 55.6%). Large single crystals (milligram sized) were obtained using a 0.1 mmol scale of reagents, a 20 mL vial, and 1/1 DMF/CH_3_CN as solvent, with the crystals allowed to form over several weeks. Anal. Calcd (found) for C_46_H_44_BN_4_O_2_MnCl_4_: C, 61.91 (61.73); H, 4.97 (4.90); N, 6.28 (6.26). IR (FT-ATR diamond anvil): ν/cm^–1^ 3234(w), 3052(w), 1650(m), 1625(s), 1578(m), 1529(w), 1478(w), 1454(m), 1427(s), 1376(s), 1285(s), 1189(m), 1174(m), 1073(m), 985(m), 863(m), 769(s), 734(s), 708(s), 610(s), 524(m), 510(m). All chemicals were purchased from Sigma-Aldrich. All other reagents were purchased from standard sources and were used as received.

### Magnetic Measurements

The magnetic properties of a polycrystalline sample of complex **1** were measured using a Quantum Design MPMS XL SQUID instrument. The molar magnetic susceptibility multiplied by the temperature (χ_M_*T*) as a function of temperature of a polycrystalline sample of complex **1** was used to follow the temperature-dependent magnetic susceptibility in cooling and warming sequences in 2 K steps between 4 and 350 K with an applied magnetic field of 0.1 T. Diamagnetic corrections were calculated using Pascal’s constants and applied to all data. The calculated χ_M_*T* vs *T* data are given in Tables S1 and S2 for cooling and warming cycles, respectively.

### Single-Crystal X-ray Diffraction

Single-crystal X-ray diffraction (SCXRD) data of complex **1** were collected at 250, 150, 115, and 82 K on a first single crystal and at 10 K on a second single crystal using an Oxford Diffraction Xcalibur3 X-ray diffractometer (Oxford Instruments, Oxford, United Kingdom) fitted with an enhanced source, using Mo Kα radiation (λ = 0.71073 Å). The diffractometer is fitted with a Sapphire3 detector. The selected order of unit cell parameters allows for a direct comparison between different phases.

Changes in unit cell volume, cell parameters, and Bragg peak intensities as a function of temperature were investigated on a new single crystal of complex **1** during cooling from 250 to 83 K and warming from 83 to 200 K (2–3 K steps with 200 K/h cooling/heating rate) by means of an Agilent Technologies SuperNova Single Source X-ray diffractometer with a microsource, using Cu Kα (λ = 1.54184 Å) radiation, fitted with an EosS2 detector.

Single crystals were mounted on cactus needles with Parabar 10312 oil.

Nitrogen flow 800Plus and 700Plus series cryostats from Oxford Cryosystems were used respectively for the variable-temperature measurements and for the full data collections. A helium flow Oxford Diffraction Helijet Cryostream was used for the helium experiments performed below 80 K. Multiple crystals were tested at 80, 40, 20, and 10 K.

The CrysAlisPRO^95^ software package from Rigaku Oxford Diffraction was used for all data collections and data processing (indexing, integration, and reduction). Full data sets were collected on assuming that the Friedel pairs are not equivalent. This allowed for sufficient data coverage for noncentrosymmetric crystallographic systems. Data scaling and absorption corrections were applied during the final data processing. All structures were solved by dual direct methods with ShelXT^96,97^ and refined by full-matrix least squares on *F*^2^ using ShelXL^97,98^ in OLEX^2^GUI.^99^ The structures are all polar and for *P*1 are also chiral. In all cases, the absolute structure was determined unambiguously from the diffraction data, as confirmed by Flack parameters (Table S3).

All non-hydrogen atoms were refined anisotropically; H atoms were constrained by geometry. When applicable, ISOR restraints were applied for occupational disorder of BPh_4_^–^ anions. For data collected at 10, 82, and 115 K, twin refinement was included due to the formation of ferroelastic twin domains associated with changes in symmetry from monoclinic to triclinic; only two main domains were considered in the final twin refinements.

Structural refinement details and parameters are summarized in Table S3. Bond length and bond angle details are provided in Table S4 with a comparison of Mn–donor bond lengths in the four different HT, INT2, INT1, and LT structural phases. Perspective views of the asymmetric unit of structures measured at 250, 150, 115, 82, and 10 K are provided in Figures S3–S7, shown with 50% atomic probability distributions for ellipsoids.

Precession images in the (*h*0*l*) plane were calculated for 250, 150, 115, and 82 K data sets of complex **1** and are shown in Figure S15.

CCDC-2100797 (250 K), CCDC-2100798 (150 K), CCDC-2100799 (115 K), CCDC-2100800 (82 K), and CCDC-2100801 (10 K) contain the crystal data collection and refinement parameter details for this paper, which can be obtained free of charge via www.ccdc.cam.ac.uk/conts/retrieving.html (or from the Cambridge Crystallographic Data Centre, 12 Union Road, Cambridge CB2 1EZ, U.K.; fax: (+44) 1223-336-033; or deposit@ccdc.ca.ac.uk).

### Resonant Ultrasound Spectroscopy

The resonant ultrasound spectroscopy (RUS) technique is commonly used to investigate phase transitions^88^ and has been used recently to follow changes in elastic and anelastic properties at both symmetry-breaking and non-symmetry-breaking transitions in SCO materials.^30,54^ A single crystal of complex **1** with an irregular shape and dimensions of around 1 mm in all directions was mounted between two piezoelectric transducers.^100^ In the Cambridge system, the piezoelectric transducers sit in a custom built RUS head, which is inserted into a helium flow cryostat.^101^ The sample chamber is filled with a few millibars of helium for reliable temperature control of the crystal. The system is set up to detect acoustic resonances of small crystals or ceramic samples in the frequency range 0.1–2 MHz. For data processing, the raw spectra were transferred to the software package Igor Pro (WaveMetrics). Fits to individual peaks using an asymmetric Lorentzian function gave the values of peak frequency, *f*, and the peak widths at half height, Δ*f*. *f*^2^ scales with the combination of elastic constants that determines each resonance mode.^100^ The inverse mechanical quality factor, *Q*^–1^, was taken as *Q*^–1^*= (*Δ*f/f*) and is a measure of acoustic loss. Individual resonances are dominated by shear motions with only relative contributions from breathing motions.

### Magnetoelectric Coupling

The electric polarization of complex **1** was measured in a 14 T Physical Properties Measurement System (PPMS) by Quantum Design with a custom probe. Silver paint was used to create capacitor plates on parallel faces of the crystal along the *c* axis and connected to Gore coaxial cables. Thermalization was provided by ^4^He gas and a sapphire plate on which the sample and a Cernox thermometer were mounted together with GE 7031 varnish and silver paint. Electric polarization was measured by a Keithley 6517A electrometer in charge mode, and the capacitance was measured at 1 kHz and 15 V with an Andeen-Hagerling 2500A capacitance bridge.

Electric polarization was also measured in millisecond capacitor-driven resistive magnets up to 65 T at the National High Magnetic Pulsed Field Facility at Los Alamos National Laboratory.^102−104^ Two single crystals were mounted on a custom-designed electric polarization probe so that the magnetic field was aligned both parallel and perpendicular to the *c* axis (the macroscopic long axis) of the single crystals at room temperature. The sapphire sample holder was equipped with a Cernox thermometer, and a minimum of GE 7031 varnish was employed to mount the sample. The faces of the single crystals were coated with silver paint to form capacitor plates. The sample was cooled in ^4^He gas between 4 and 150 K. The electric polarization in pulsed magnetic fields was measured by recording the change in surface charge using a Stanford Research 570 current to voltage converter when a pulsed magnetic field was applied up to 65 T.^105,106^ The pulsed field magnetization was determined by an inductive technique in compensated coils^107^ with sample-in sample-out background subtraction. The sample was mounted in the interior of a plastic capsule using Apiezon vacuum grease. Where not otherwise marked, measurements were conducted on one single crystal for magnetic and electric fields along the *c* axis. *T* was controlled using ^4^He gas or liquid via a Cernox thermometer mounted near the magnetization sample or on sapphire together with the polarization sample. Throughout, standard errors are smaller than line widths and point sizes when not shown.

## Supporting Information Available

Details of the magnetometry measurements, single-crystal X-ray diffraction data, resonant ultrasound spectroscopy methodology, and calculations (PDF)

## Accession Codes

CCDC 2100797–2100801 contain the supplementary crystallographic data for this paper. These data can be obtained free of charge via www.ccdc.cam.ac.uk/data_request/cif, or by emailing data_request@ccdc.cam.ac.uk, or by contacting The Cambridge Crystallographic Data Centre, 12 Union Road, Cambridge CB2 1EZ, UK; fax: +44 1223 336033.

## Notes

A report on the magnetic and dielectric properties of [Mn^III^(3,5-diCl-sal_2_(323))]BPh_4_ together with crystallographic data for three of the four structural phases has been published during the revision process for this article, see ref (108).
