# Supplementary material for: Domain Wall Dynamics in a Ferroelastic Spin Crossover Complex with Giant Magnetoelectric Coupling
Source: J Am Chem Soc. 2021 Dec 23;144(1):195–211. doi: 10.1021/jacs.1c08214 (PMC8759087; doi:10.1021/jacs.1c08214)
Supplement: Supplementary file 1 — ja1c08214_si_001.pdf [file ja1c08214_si_001.pdf]

Supporting Information for

Domain Wall Dynamics in a Ferroelastic Spin Crossover Complex with Giant Magnetoelectric Coupling

Vibe Boel Jakobsen,[†] Elzbieta Trzop,[‡] Emiel Dobbelaar,[†,#] Laurence C. Gavin,[†] Shalinee Chikara,[§,«] Xiaxin Ding, [||,¤] Minseong Lee, [||] Kane Esien,[⊥] Helge Müller-Bunz,[†] Solveig Felton,[⊥] Eric Collet,[*‡] Michael A. Carpenter,[*∇] Vivien S. Zapf,[*,||] and Grace G. Morgan[*,†]

[†] School of Chemistry, University College Dublin, Belfield, Dublin 4, Ireland

[‡] Univ Rennes, CNRS, IPR (Institut de Physique de Rennes) - UMR 6251, F-35000 Rennes, France

[§] Department of Physics, Auburn University Auburn, AL 36849, United States

[||] National High Magnetic Field Laboratory, Los Alamos National Laboratory, Los Alamos, New Mexico 87545, United States

[⊥] Centre for Nanostructured Media, School of Mathematics and Physics, Queen's University of Belfast, Belfast, BT7 1NN, Northern Ireland, United Kingdom

[∇] Department of Earth Sciences, University of Cambridge, Downing Street, Cambridge CB2 3EQ, England, United Kingdom

Table of contents

S1 Magnetometry measurements
S2 Single crystal X-ray diffraction
S3 Resonant ultrasound spectroscopy
S4 Magnetoelectric coupling
S5 References

S1 Magnetometry measurements

Table S1 Magnetic data for polycrystalline sample of complex **1** in cooling mode.

Cooling mode									
**Tempe-rature (K)**	**$\chi_M T$ (cm^{3}K mol^{-1})**	**Tempe-rature (K)**	**$\chi_M T$ (cm^{3}K mol^{-1})**	**Tempe-rature (K)**	**$\chi_M T$ (cm^{3}K mol^{-1})**	**Tempe-rature (K)**	**$\chi_M T$ (cm^{3}K mol^{-1})**	**Tempe-rature (K)**	**$\chi_M T$ (cm^{3}K mol^{-1})**
350.045	2.6348	273.605	2.6792	197.614	2.7462	121.705	2.3009	46.011	0.9935
347.508	2.6312	271.603	2.6797	195.627	2.7495	119.710	2.2886	44.004	0.9909
345.550	2.6057	269.596	2.6829	193.623	2.7512	117.701	2.2758	41.998	0.9874
343.561	2.6090	267.583	2.6841	191.609	2.7529	115.708	2.2638	39.991	0.9841
341.565	2.6111	265.586	2.6863	189.623	2.7550	113.706	2.2511	37.990	0.9802
339.573	2.6136	263.614	2.6881	187.633	2.7564	111.721	2.2394	35.985	0.9755
337.577	2.6158	261.566	2.6908	185.631	2.7590	109.721	2.2269	33.985	0.9704
335.574	2.6182	259.583	2.6929	183.635	2.7594	107.727	2.2150	31.987	0.9657
333.616	2.6210	257.593	2.6958	181.633	2.7625	105.734	2.2023	29.983	0.9583
331.602	2.6225	255.591	2.6974	179.633	2.7637	103.734	2.1899	27.985	0.9487
329.582	2.6248	253.597	2.6988	177.637	2.7656	101.737	2.1781	25.986	0.9373
327.623	2.6277	251.599	2.7013	175.640	2.7682	99.748	2.1665	23.991	0.9229
325.601	2.6300	249.585	2.7023	173.634	2.7695	97.743	2.1551	22.078	0.9052
323.602	2.6304	247.599	2.7040	171.634	2.7714	95.777	2.1440	20.057	0.8822
321.593	2.6334	245.593	2.7066	169.640	2.7722	93.777	2.1322	18.040	0.8522
319.615	2.6362	243.588	2.7072	167.646	2.7742	91.768	2.1219	16.029	0.8139
317.608	2.6377	241.615	2.7104	165.647	2.7753	89.767	2.1108	14.022	0.7643
315.603	2.6395	239.595	2.7119	163.651	2.7770	87.809	2.1003	12.014	0.6989
313.597	2.6423	237.589	2.7134	161.644	2.7777	85.829	2.0891	10.010	0.6172
311.600	2.6439	235.600	2.7155	159.661	2.7790	83.830	2.0656	8.010	0.5161
309.612	2.6444	233.599	2.7174	157.653	2.7789	82.063	1.5338	6.009	0.3976
307.586	2.6472	231.596	2.7194	155.654	2.7808	80.054	1.1661	3.995	0.2697
305.610	2.6487	229.590	2.7206	153.653	2.7811	78.058	1.1211		
303.605	2.6502	227.609	2.7213	151.659	2.7825	76.062	1.1000		
301.612	2.6524	225.591	2.7234	149.662	2.7832	74.062	1.0828		
299.594	2.6541	223.594	2.7252	147.669	2.7843	72.062	1.0686		
297.597	2.6555	221.602	2.7266	145.663	2.7854	70.060	1.0563		
295.609	2.6580	219.614	2.7280	143.657	2.7856	68.064	1.0462		
293.605	2.6595	217.605	2.7289	141.671	2.7869	66.085	1.0389		
291.593	2.6610	215.602	2.7303	139.675	2.7839	64.071	1.0314		
289.575	2.6631	213.602	2.7319	137.680	2.4306	62.071	1.0250		
287.611	2.6646	211.596	2.7334	135.669	2.3857	60.081	1.0198		
285.613	2.6667	209.607	2.7348	133.688	2.3732	58.091	1.0148		
283.603	2.6684	207.611	2.7361	131.679	2.3609	56.073	1.0104		
281.613	2.6711	205.612	2.7387	129.694	2.3485	54.074	1.0063		
279.602	2.6733	203.616	2.7392	127.690	2.3359	52.057	1.0028		
277.594	2.6743	201.618	2.7428	125.691	2.3239	50.040	0.9997		
275.595	2.6772	199.608	2.7456	123.699	2.3134	48.015	0.9968		

Table S2 Magnetic data for polycrystalline sample of complex **1** in warming mode.

**Warming mode**									
**Tempe-rature (K)**	**$\chi_M T$ (cm^{3}K mol^{-1})**	**Tempe-rature (K)**	**$\chi_M T$ (cm^{3}K mol^{-1})**	**Tempe-rature (K)**	**$\chi_M T$ (cm^{3}K mol^{-1})**	**Tempe-rature (K)**	**$\chi_M T$ (cm^{3}K mol^{-1})**	**Tempe-rature (K)**	**$\chi_M T$ (cm^{3}K mol^{-1})**
3.997	0.2698	80.124	1.1388	156.246	2.7953	232.308	2.7220	308.351	2.6449
6.003	0.3973	82.120	1.1693	158.258	2.7939	234.303	2.7215	310.343	2.6431
8.004	0.5159	84.129	1.2229	160.265	2.7932	236.305	2.7202	312.353	2.6409
10.001	0.6175	86.134	1.3806	162.261	2.7930	238.307	2.7180	314.368	2.6407
11.998	0.6986	88.152	1.8192	164.257	2.7920	240.315	2.7162	316.347	2.6381
14.008	0.7636	90.148	2.0491	166.265	2.7904	242.320	2.7134	318.367	2.6363
15.989	0.8130	92.157	2.1065	168.267	2.7896	244.320	2.7120	320.355	2.6333
17.989	0.8510	94.167	2.1311	170.266	2.7883	246.317	2.7094	322.344	2.6320
20.009	0.8812	96.166	2.1460	172.271	2.7876	248.308	2.7078	324.349	2.6296
21.994	0.9043	98.161	2.1583	174.279	2.7853	250.321	2.7053	326.345	2.6275
23.995	0.9225	100.173	2.1708	176.276	2.7841	252.316	2.7027	328.345	2.6249
26.000	0.9370	102.181	2.1832	178.278	2.7822	254.323	2.7000	330.337	2.6228
28.000	0.9486	104.180	2.1957	180.275	2.7813	256.326	2.6976	332.341	2.6225
29.999	0.9577	106.192	2.2090	182.276	2.7786	258.326	2.6955	334.324	2.6210
32.000	0.9653	108.183	2.2208	184.275	2.7766	260.320	2.6941	336.339	2.6174
34.000	0.9715	110.194	2.2335	186.281	2.7747	262.323	2.6924	338.346	2.6166
36.001	0.9767	112.189	2.2466	188.285	2.7726	264.351	2.6900	340.342	2.6145
38.002	0.9811	114.205	2.2588	190.285	2.7694	266.333	2.6872	342.356	2.6133
40.005	0.9851	116.193	2.2714	192.288	2.7679	268.323	2.6854	344.347	2.6110
42.007	0.9884	118.198	2.2838	194.293	2.7665	270.322	2.6826	346.333	2.6100
44.008	0.9916	120.191	2.2964	196.292	2.7645	272.325	2.6812	348.342	2.6087
46.012	0.9948	122.240	2.3097	198.279	2.7622	274.326	2.6797	350.334	2.6074
48.016	0.9976	124.238	2.3222	200.292	2.7600	276.331	2.6766		
50.030	1.0005	126.211	2.3339	202.293	2.7589	278.319	2.6750		
52.036	1.0034	128.200	2.3452	204.306	2.7561	280.334	2.6740		
54.047	1.0066	130.202	2.3573	206.292	2.7548	282.349	2.6714		
56.052	1.0100	132.210	2.3689	208.297	2.7521	284.326	2.6676		
58.055	1.0144	134.210	2.3817	210.299	2.7498	286.325	2.6658		
60.061	1.0185	136.204	2.3928	212.305	2.7481	288.329	2.6647		
62.066	1.0239	138.237	2.4070	214.303	2.7466	290.338	2.6619		
64.075	1.0300	140.227	2.5091	216.297	2.7437	292.325	2.6611		
66.081	1.0373	142.227	2.8015	218.303	2.7423	294.329	2.6590		
68.080	1.0455	144.235	2.7999	220.310	2.7394	296.333	2.6570		
70.094	1.0555	146.235	2.7976	222.304	2.7370	298.335	2.6557		
72.094	1.0671	148.254	2.7985	224.305	2.7352	300.343	2.6543		
74.107	1.0811	150.255	2.7973	226.313	2.7355	302.334	2.6527		
76.103	1.0966	152.266	2.7966	228.306	2.7318	304.330	2.6496		
78.106	1.1160	154.216	2.7948	230.318	2.7290	306.345	2.6473		

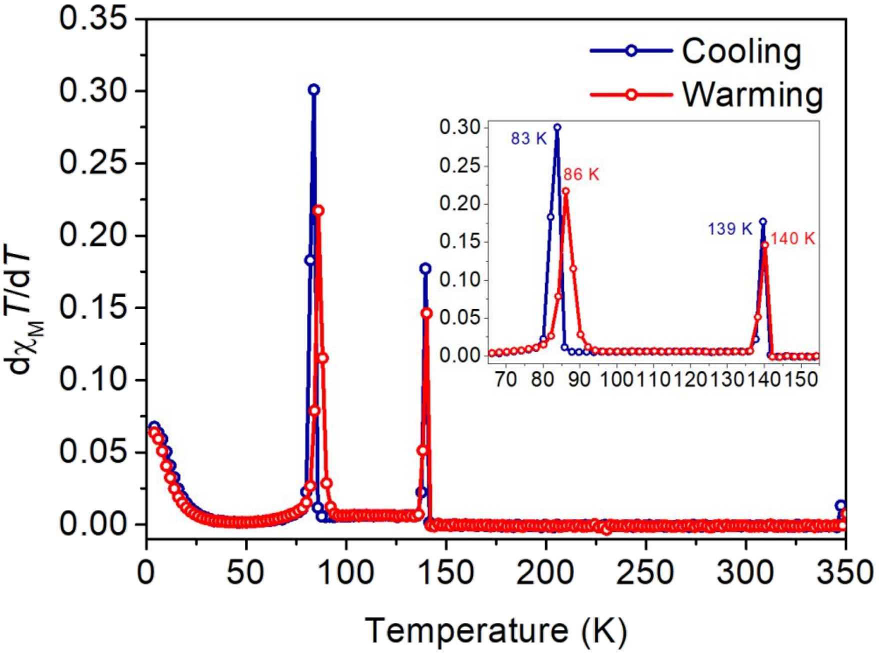

Figure S1 Derivative of $\chi_M T$ versus temperature, T for complex **1** showing discontinuities. The inset shows $d\chi_M T/T$ maximum values at 83 K (cooling) and 86 K (heating) in the lower hysteresis region and 139 K (cooling) and 140 K (heating) in the upper hysteresis window.

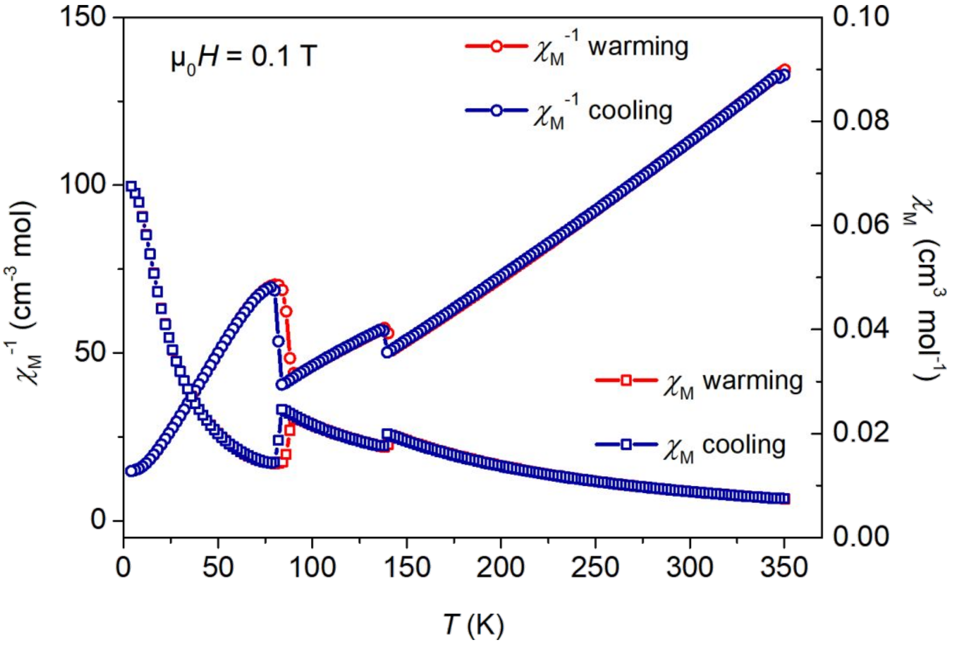

Figure S2 Temperature dependence of χ_M^{-1} (left axis) and χ_M (right axis) in warming and cooling modes for complex **1**.

S2 Single crystal X-ray diffraction

S2.1 Crystal and structure refinement data

Table S3 Crystallographic data for complex **1** at 10 K, 82 K, 115 K, 150 K and 250 K.

	**1,** 10 K	**1,** 82 K	**1,** 115 K	**1,** 150 K	**1,** 250 K
CCDC number	2100801	2100800	2100799	2100798	2100797
Empirical formula	$C_{46}H_{44}BCl_4MnN_4O_2$	$C_{46}H_{44}BCl_4MnN_4O_2$	$C_{46}H_{44}BCl_4MnN_4O_2$	$C_{46}H_{44}BCl_4MnN_4O_2$	$C_{46}H_{44}BCl_4MnN_4O_2$
Molecular formula	$[C_{22}H_{24}N_4O_2MnCl_4]^+$ $[C_{24}H_{20}B]^-$	$[C_{22}H_{24}N_4O_2MnCl_4]^+$ $[C_{24}H_{20}B]^-$	$[C_{22}H_{24}N_4O_2MnCl_4]^+$ $[C_{24}H_{20}B]^-$	$[C_{22}H_{24}N_4O_2MnCl_4]^+$ $[C_{24}H_{20}B]^-$	$[C_{22}H_{24}N_4O_2MnCl_4]^+$ $[C_{24}H_{20}B]^-$
Formula weight	892.40	892.40	892.40	892.40	892.40
Wavelength	0.71073	0.71073	0.71073	0.71073	0.71073
Crystal system	Triclinic	Triclinic	Triclinic	Monoclinic	Monoclinic
Space group	P1	P1	P1	Pc	Cc
a (Å)	14.2889(4)	14.4026(7)	14.2241(4)	14.7537(3)	14.8473(5)
b (Å)	13.2527(7)	13.3973(7)	22.9492(6)	22.6797(3)	22.6658(5)
c (Å)	13.8907(8)	13.9646(7)	13.4248(4)	14.2596(3)	14.4182(5)
α (°)	117.134(6)	117.044(5)	94.753(2)	90	90
β (°)	104.637(4)	104.573(4)	103.722(2)	117.133(3)	117.027(4)
γ (°)	104.078(4)	104.062(4)	80.125(2)	90	90
Volume ($Å^3$)	2059.3(2)	2113.7(2)	4190.1(2)	4246.30(17)	4322.2(3)
Z	2	2	4	4	4
Density (calculated) (mg/m^3)	1.439	1.402	1.415	1.396	1.371
Absorption coefficient (mm^{-1})	0.625	0.609	0.615	0.607	0.596
Crystal size (mm^3)	0.243 × 0.151 × 0.124	0.298 × 0.258 × 0.133	0.328× 0.235 × 0.132	0.334 × 0.181 × 0.152	0.334 × 0.181 × 0.152
Goodness–of–fit on F^2	1.004	0.942	0.800	1.014	1.032
Final R indices [I>2sigma(I)]	R_1 = 0.0637, wR_2 = 0.1462	R_1 = 0.0529, wR_2 = 0.1145	R_1 = 0.0389, wR_2 = 0.0692	R_1 = 0.0379, wR_2 = 00746	R_1 = 0.0388, wR_2 = 0.0781
Absolute structure parameter	-0.16(3)	0.042(17)	0.023(10)	-0.007(6)	0.008(9)

Table S4 Selected bond lengths (Å) and angles (°) for complex **1**.

	**1** (10 K)	**1** (82 K)	**1** (115 K)	**1** (150 K)	**1** (250 K)
			**Bond lengths (Å)**		
Mn1-O1$_{phen}$	1.864(10)	1.883(5)	1.871(4)	1.871(2)	1.869(3)
Mn1-O2$_{phen}$	1.883(10)	1.897(5)	1.881(4)	1.872(2)	1.872(3)
Mn1-N1$_{imine}$	2.001(12)	2.011(6)	2.017(5)	2.125(3)	2.115(3)
Mn1-N2$_{amine}$	2.067(11)	2.102(6)	2.120(5)	2.217(3)	2.225(3)
Mn1-N3$_{amine}$	2.040(12)	2.072(6)	2.073(5)	2.240(3)	2.216(3)
Mn1-N4$_{imine}$	1.967(11)	2.007(7)	2.013(6)	2.079(3)	2.088(3)
Mn2-O3$_{phen}$	1.876(10)	1.880(5)	1.874(4)	1.866(2)	
Mn2-O4$_{phen}$	1.877(10)	1.902(5)	1.880(4)	1.875(2)	
Mn2-N5$_{imine}$	1.987(12)	2.001(7)	2.034(5)	2.107(3)	
Mn2-N6$_{amine}$	2.042(12)	2.068(6)	2.141(6)	2.234(3)	
Mn2-N7$_{amine}$	2.089(11)	2.096(6)	2.107(6)	2.204(3)	
Mn2-N8$_{imine}$	1.999(12)	2.020(6)	2.027(6)	2.090(3)	
Mn3-O5$_{phen}$			1.864(4)		
Mn3-O6$_{phen}$			1.875(4)		
Mn3-N9$_{imine}$			2.106(5)		
Mn3-N10$_{amine}$			2.242(5)		
Mn3-N11$_{amine}$			2.206(5)		
Mn3-N12$_{imine}$			2.081(5)		
Mn4-O7$_{phen}$			1.864(4)		
Mn4-O8$_{phen}$			1.876(4)		
Mn4-N13$_{imine}$			2.101(6)		
Mn4-N14$_{amine}$			2.239(7)		
Mn4-N15$_{amine}$			2.229(7)		
Mn4-N16$_{imine}$			2.091(5)		
			**Bond angles (°)**		
O1-Mn1-O2	176.0(4)	176.0(2)	176.74(19)	177.03(11)	176.74(13)
O1-Mn1-N1	89.7(4)	89.7(2)	89.1(2)	87.36(11)	87.52(12)
O1-Mn1-N2	94.1(4)	93.4(2)	93.0(2)	93.84(12)	93.57(13)
O1-Mn1-N3	85.0(4)	85.2(2)	85.2(2)	85.03(12)	85.97(13)
O1-Mn1-N4	89.4(4)	89.7(2)	90.4(2)	90.04(11)	90.15(12)
O2-Mn1-N1	94.1(4)	94.1(2)	94.01(19)	92.78(11)	92.96(12)
O2-Mn1-N2	87.5(4)	88.2(2)	88.17(19)	89.12(11)	89.68(13)
O2-Mn1-N3	91.5(4)	91.3(2)	91.9(2)	95.81(12)	94.58(14)
O2-Mn1-N4	88.6(4)	88.3(2)	88.0(2)	87.18(11)	86.67(12)
N1-Mn1-N2	86.7(4)	86.1(2)	85.6(2)	82.05(13)	82.74(14)
N1-Mn1-N3	169.8(5)	168.7(3)	167.6(2)	159.22(12)	160.58(13)
N2-Mn1-N3	85.1(4)	84.2(3)	83.7(2)	79.21(14)	79.43(16)
N4-Mn1-N1	98.8(5)	100.2(3)	102.8(2)	113.27(12)	111.50(12)

N4-Mn1-N2	173.5(5)	173.0(2)	171.0(2)	164.39(13)	165.45(13)
N4-Mn1-N3	89.8(5)	89.8(3)	88.2(2)	86.08(12)	86.81(14)
O3-Mn2-O4	174.8(5)	175.8(2)	176.3(2)	176.32(11)	
O3-Mn2-N5	90.8(5)	90.7(3)	89.5(2)	88.22(11)	
O3-Mn2-N6	83.8(5)	84.5(2)	94.4(2)	94.04(11)	
O3-Mn2-N7	92.6(5)	92.8(3)	84.6(2)	85.60(11)	
O3-Mn2-N8	90.6(5)	89.8(2)	90.4(2)	89.76(11)	
O4-Mn2-N5	88.6(5)	88.5(3)	94.0(2)	93.11(11)	
O4-Mn2-N6	91.1(5)	91.3(2)	87.2(2)	89.53(11)	
O4-Mn2-N7	87.4(4)	87.5(2)	92.2(2)	94.19(11)	
O4-Mn2-N8	94.6(5)	94.4(2)	87.5(2)	86.56(11)	
N5-Mn2-N6	89.3(5)	88.9(3)	84.0(2)	82.83(11)	
N5-Mn2-N7	172.6(5)	172.7(3)	164.9(2)	160.85(11)	
N7-Mn2-N6	100.1(5)	100.4(3)	82.5(2)	79.55(11)	
N8-Mn2-N5	84.6(5)	85.0(3)	105.9(2)	111.04(11)	
N8-Mn2-N6	169.1(5)	169.2(3)	169.0(2)	165.75(11)	
N8-Mn2-N7	86.4(5)	86.1(3)	88.1(2)	87.07(11)	
O5-Mn3-O6			176.1(2)		
O5-Mn3-N9			88.0(2)		
O5-Mn3-N10			94.35(19)		
O5-Mn3-N11			85.09(19)		
O5-Mn3-N12			89.8(2)		
O6-Mn3-N9			92.79(19)		
O6-Mn3-N10			89.57(19)		
O6-Mn3-N11			95.4(2)		
O6-Mn3-N12			86.3(2)		
N9-Mn3-N10			82.3(2)		
N9-Mn3-N11			159.5(2)		
N11-Mn3-N10			79.0(2)		
N12-Mn3-N9			111.9(2)		
N12-Mn3-N10			165.3(2)		
N12-Mn3-N11			87.3(2)		
O7-Mn4-O8			176.8(2)		
O7-Mn4-N13			87.8(2)		
O7-Mn4-N14			95.0(2)		
O7-Mn4-N15			84.9(2)		
O7-Mn4-N16			90.6(2)		
O8-Mn4-N13			93.3(2)		
O8-Mn4-N14			88.2(2)		
O8-Mn4-N15			95.1(2)		
O8-Mn4-N16			86.2(2)		
N13-Mn4-N14			81.9(3)		

N13-Mn4-N15	158.6(2)
N15-Mn4-N14	78.7(3)
N16-Mn4-N13	114.3(2)
N16-Mn4-N14	163.1(2)
N16-Mn4-N15	86.0(2)

S2.2 Crystal structure and packing of complex **1**

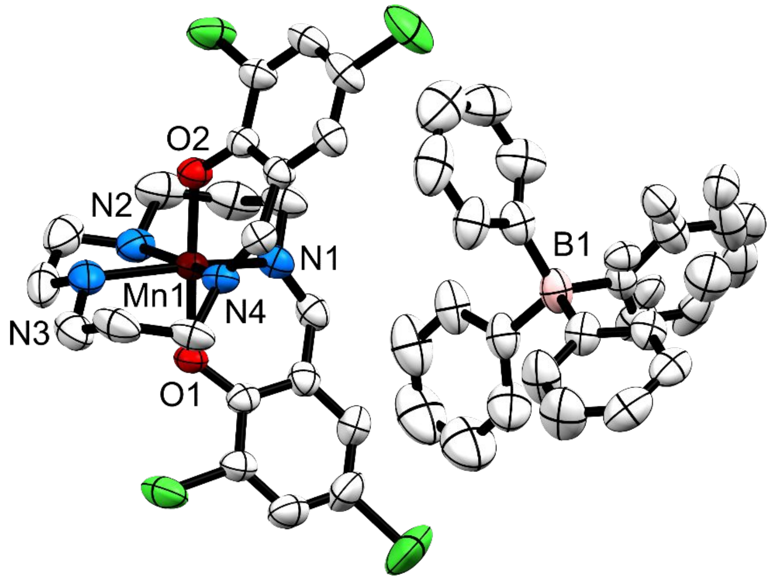

Figure S3 Asymmetric unit of complex **1**, [Mn(3,5-Cl-sal$_2$(323))]BPh$_4$ measured at 250 K shown with 50 % atomic probability distributions for ellipsoids with hydrogen atoms omitted for clarity.

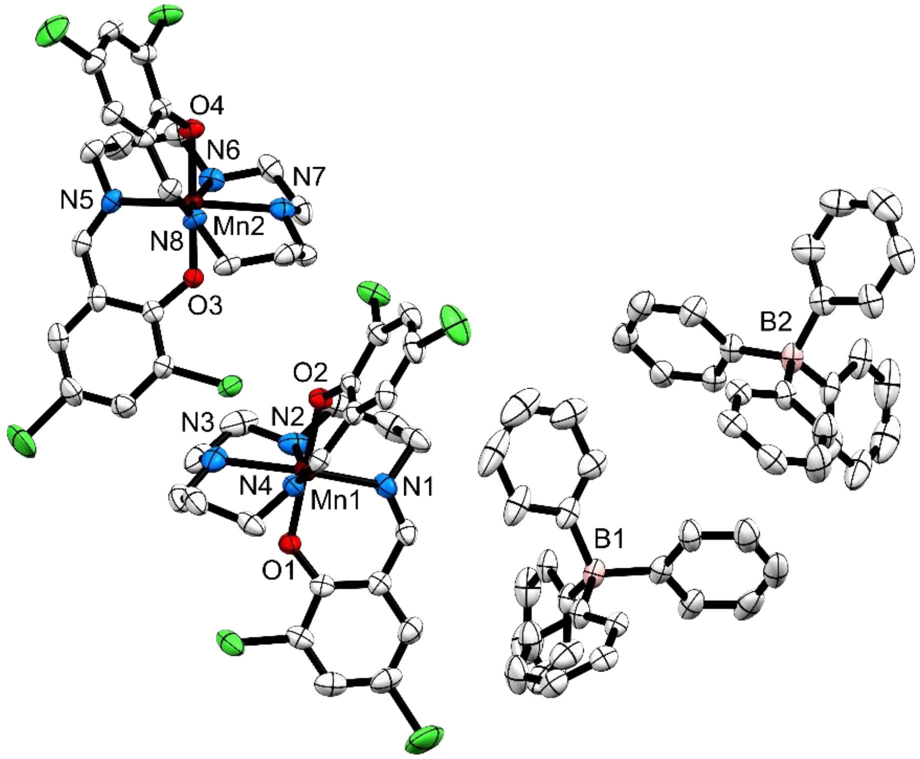

Figure S4 Asymmetric unit of complex **1**, [Mn(3,5-Cl-sal$_2$(323))]BPh$_4$ measured at 150 K shown with 50 % atomic probability distributions for ellipsoids with hydrogen atoms omitted for clarity.

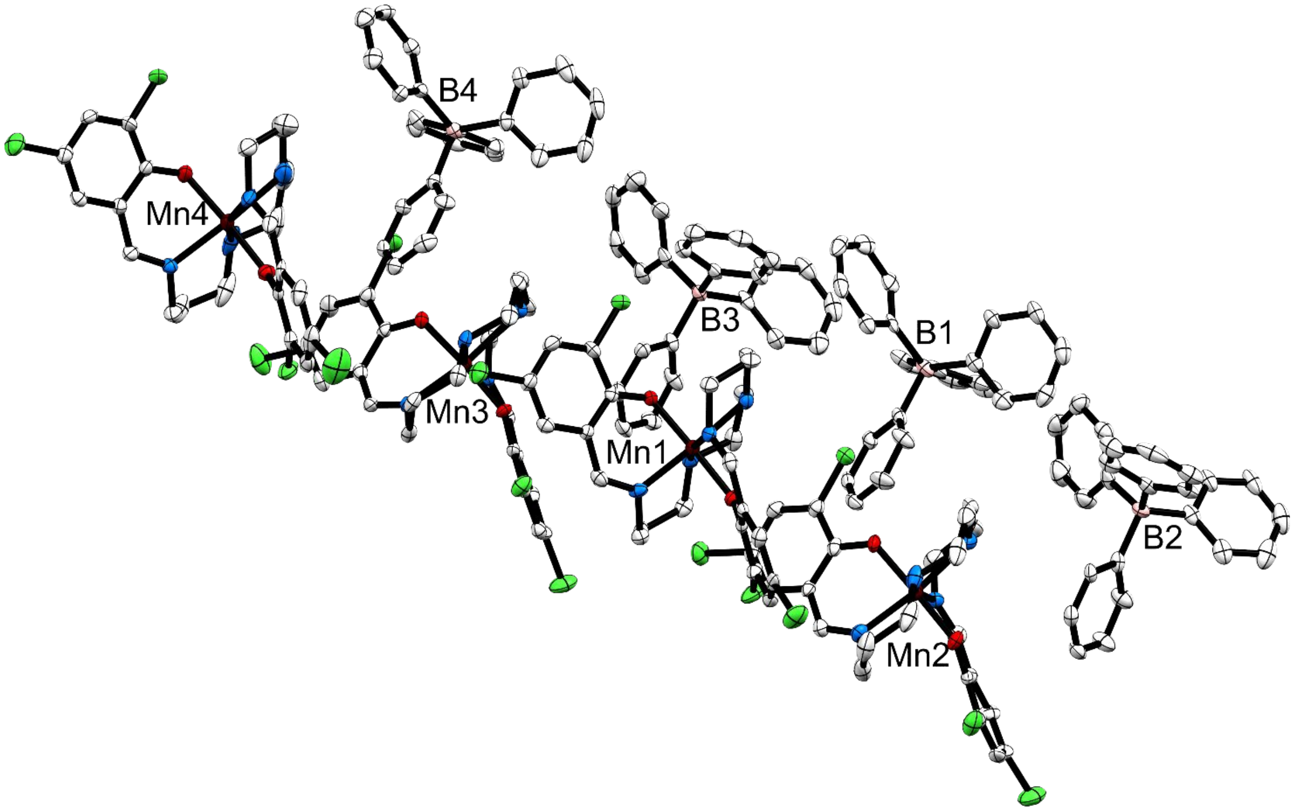

Figure S5 Asymmetric unit of complex **1**, [Mn(3,5-diCl-sal)$_2$323]BPh$_4$ measured at 115 K shown with 50 % atomic probability distributions for ellipsoids with hydrogen atoms omitted for clarity.

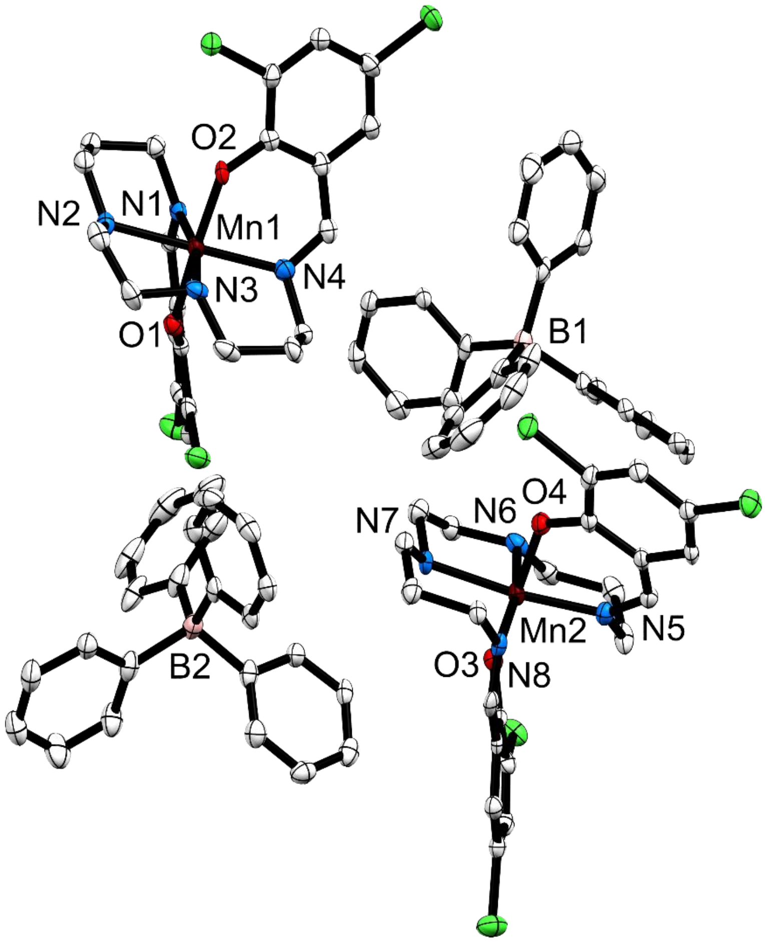

Figure S6 Asymmetric unit of complex **1**, [Mn(3,5-Cl-sal$_2$(323))]BPh$_4$ measured at 82 K shown with 50 % atomic probability distributions for ellipsoids with hydrogen atoms omitted for clarity.

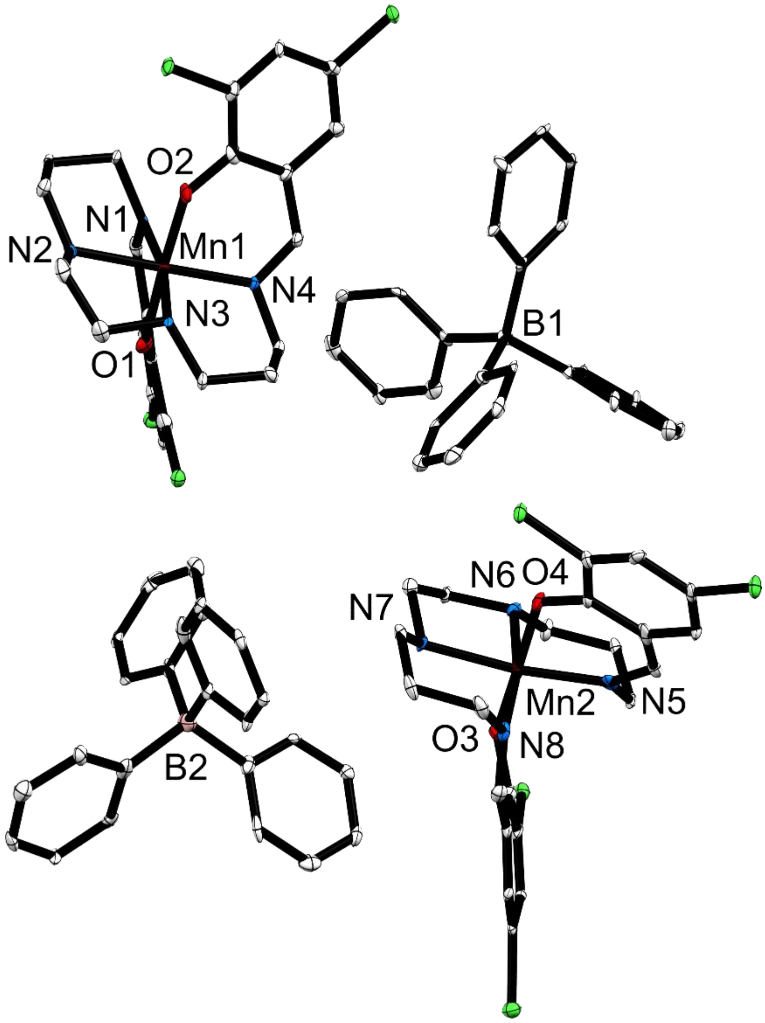

Figure S7 Asymmetric unit of complex **1**, [Mn(3,5-Cl-sal$_2$(323))]BPh$_4$ measured at 10 K shown with 50 % atomic probability distributions for ellipsoids with hydrogen atoms omitted for clarity.

Complex **1** is refined in the non-centrosymmetric polar space group *Cc* at 250 K. At 150 K, complex **1** is refined in the non-centrosymmetric polar space group *Pc* and at 115 K, 82 K and 10 K in the non-centrosymmetric chiral and polar space group *P1*. Complex **1** comprises the [Mn(3,5-diCl-sal$_2$(323))]$^+$ cation and the tetraphenylborate counteranion, BPh$_4^-$. Each unit cell in the HT, INT2 and INT1 phases contains four Mn^{3+} cations and four BPh$_4^-$ anions. Hence, the *Cc* structures have Z = 4 and Z' = 1, the *Pc* structure has Z = 4 and Z' = 2 and the *P1* structure has Z = 4 and Z' = 4. The LT *P1* structure contains two Mn^{3+} cations and two BPh$_4^-$ anions in the unit cell which results in Z = 2 and Z' = 2. The structures are all polar (with the *P1* structure also being chiral) with Flack parameters[9] of each structure being close to zero. No solvent molecules were located in any of the structures.

S2.3 Hirshfeld surface mapping

The Hirshfeld surfaces are mapped with d_{norm}, and 2D fingerprint plots were generated using CrystalExplorer 17.5.[1] The graphical plot uses a red-white-blue colour scheme for the molecular Hirshfeld surfaces. The red highlights on the Hirshfeld surfaces show contacts shorter than the van der Waals distance, contacts within the van der Waals distance are shown in white and longer contacts are shown in blue.

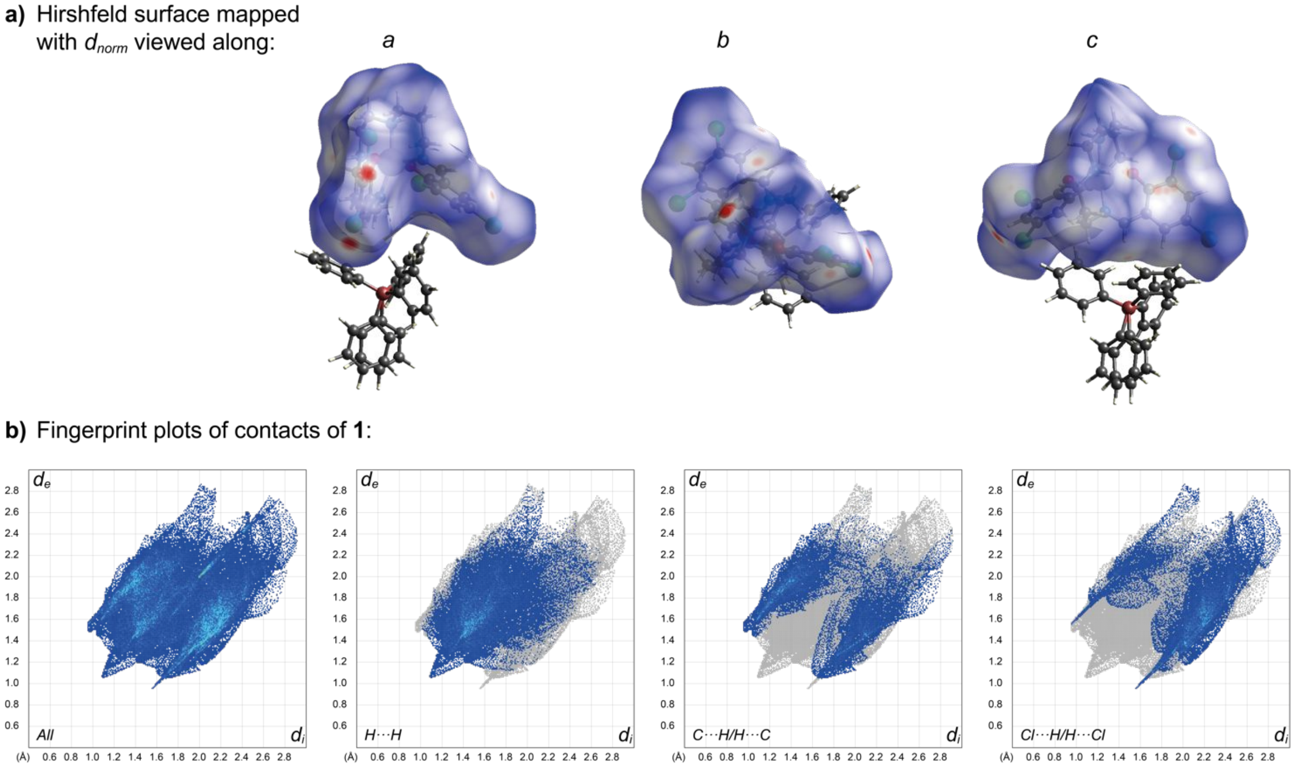

Figure S8 a) Hirshfeld surface mapped with d_{norm} for the structure of complex **1** measured at 250 K viewed along the *a*, *b* and *c* direction, b) fingerprint plots with all intermolecular interactions resolved into the contribution of H···Cl/Cl···H, H···C/C···H and H···H/H···H contacts of complex **1** at 250 K.

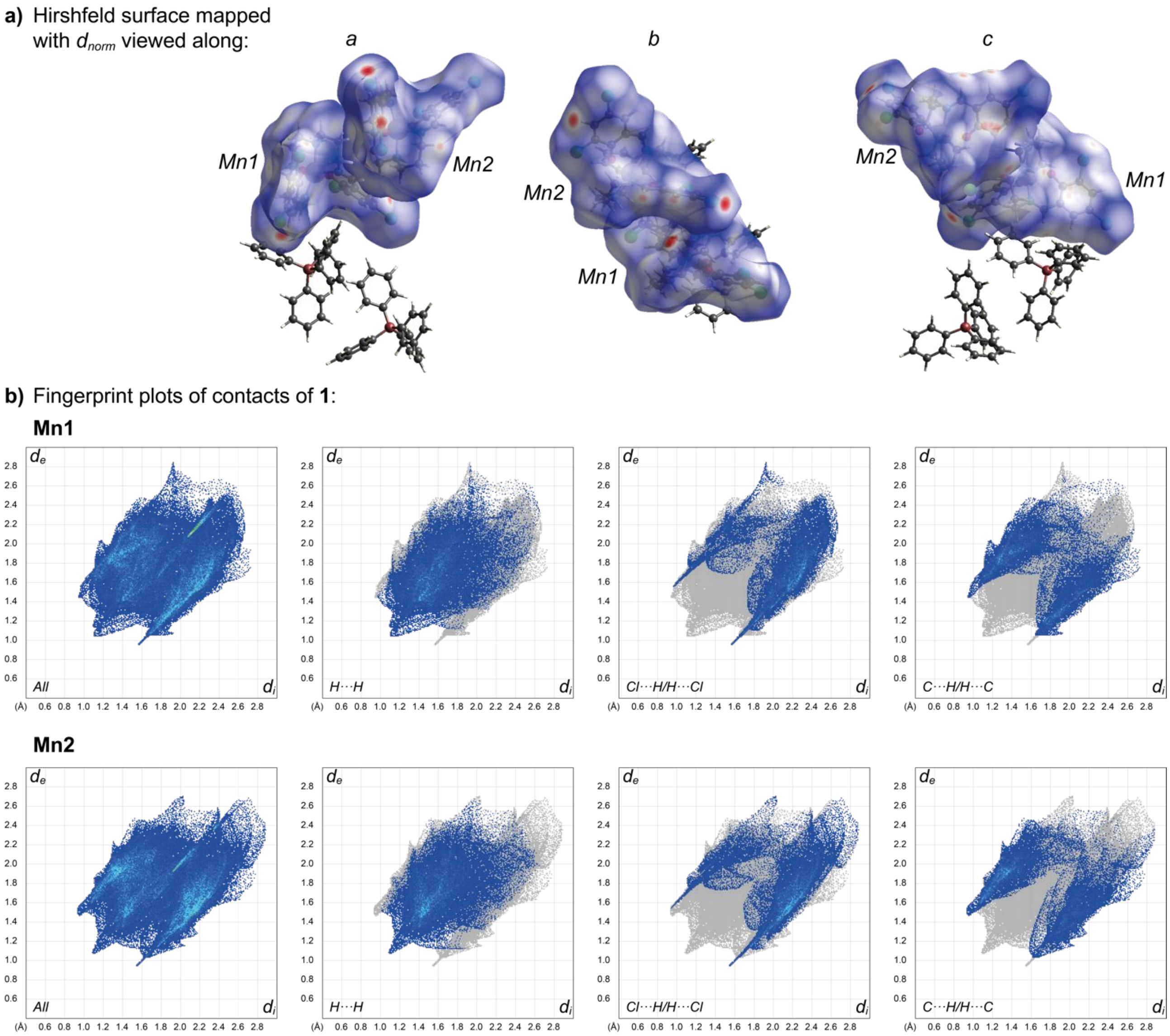

Figure S9 a) Hirshfeld surface mapped with d_{norm} for the structure of complex **1** measured at 150 K viewed along the *a*, *b* and *c* direction, b) fingerprint plots with all intermolecular interactions resolved into the contribution of H···Cl/Cl···H, H···C/C···H and H···H/H···H contacts of complex **1** at 150 K.

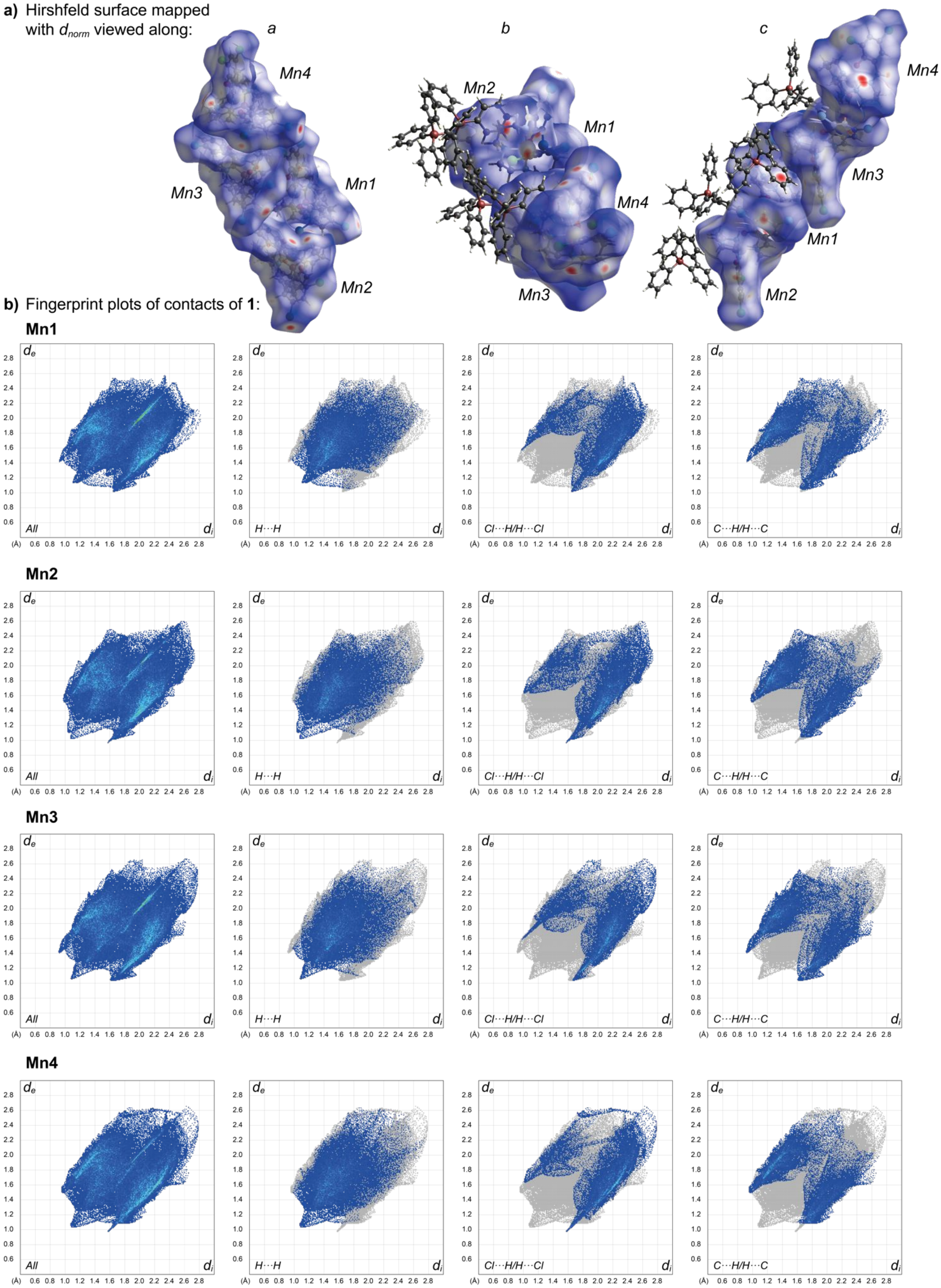

Figure S10 a) Hirshfeld surface mapped with d_{norm} for the structure of complex **1** measured at 115 K viewed along the *a*, *b* and *c* direction, b) fingerprint plots with all intermolecular interactions resolved into the contribution of H···Cl/Cl···H, H···C/C···H and H···H/H···H contacts of complex **1** at 115 K.

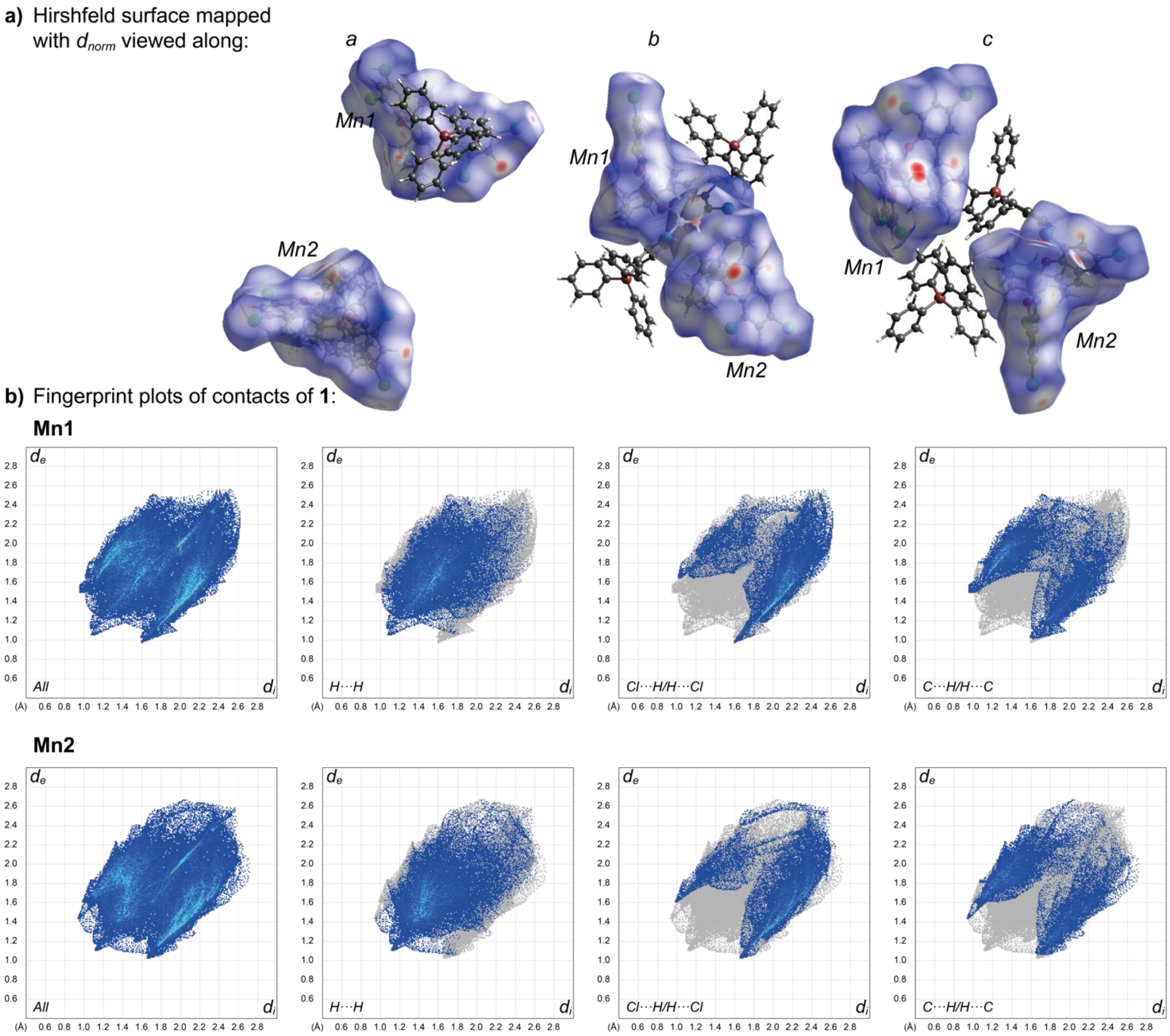

Figure S11 a) Hirshfeld surface mapped with d_{norm} for the structure of complex **1** measured at 82 K viewed along the *a*, *b* and *c* direction, b) fingerprint plots with all intermolecular interactions resolved into the contribution of H···Cl/Cl···H, H···C/C···H and H···H/H···H contacts of complex **1** at 82 K.

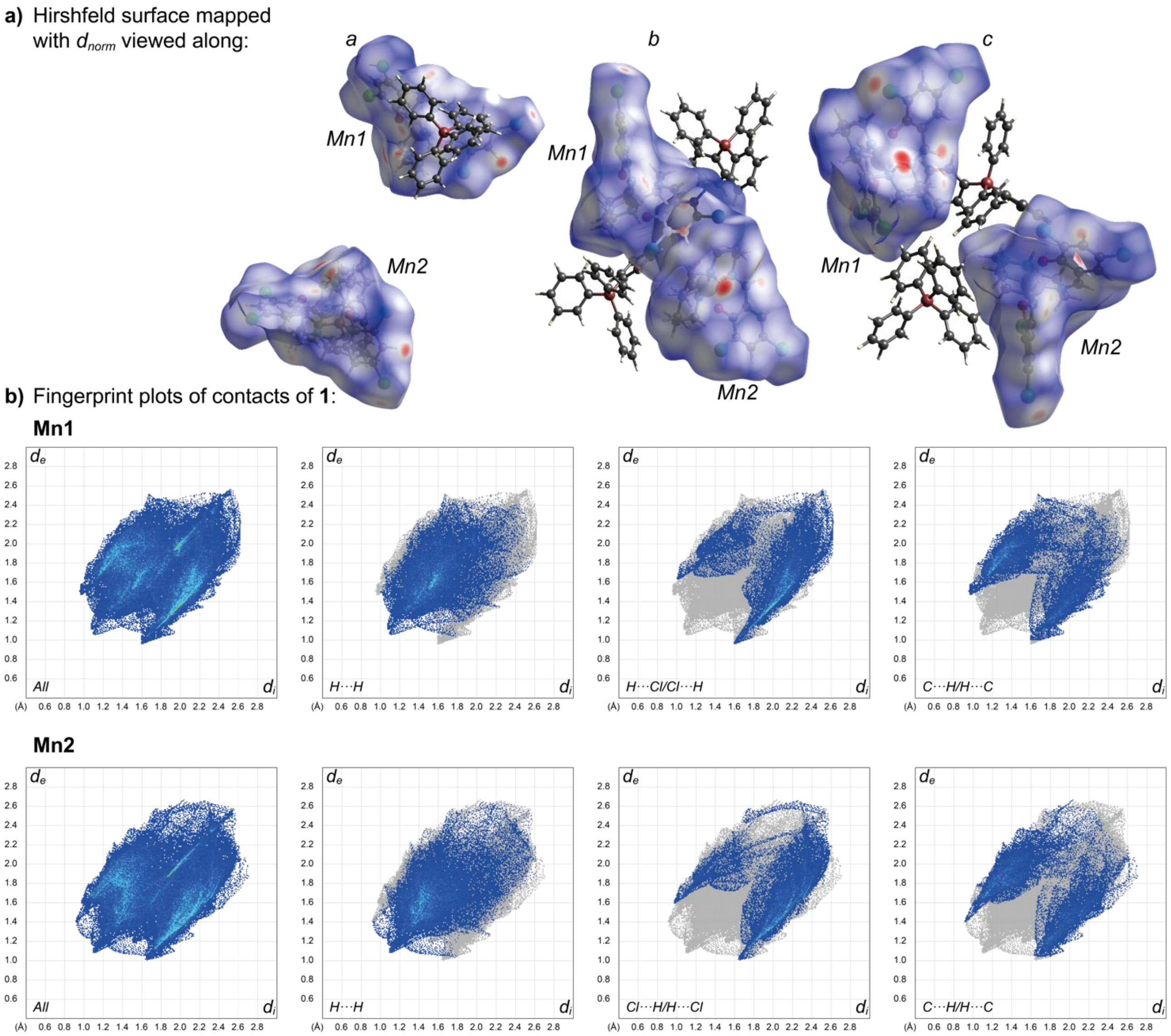

Figure S12 a) Hirshfeld surface mapped with d_{norm} for the structure of complex **1** measured at 10 K viewed along the *a*, *b* and *c* direction, b) fingerprint plots with all intermolecular interactions resolved into the contribution of H···Cl/Cl···H, H···C/C···H and H···H/H···H contacts of complex **1** at 10 K.

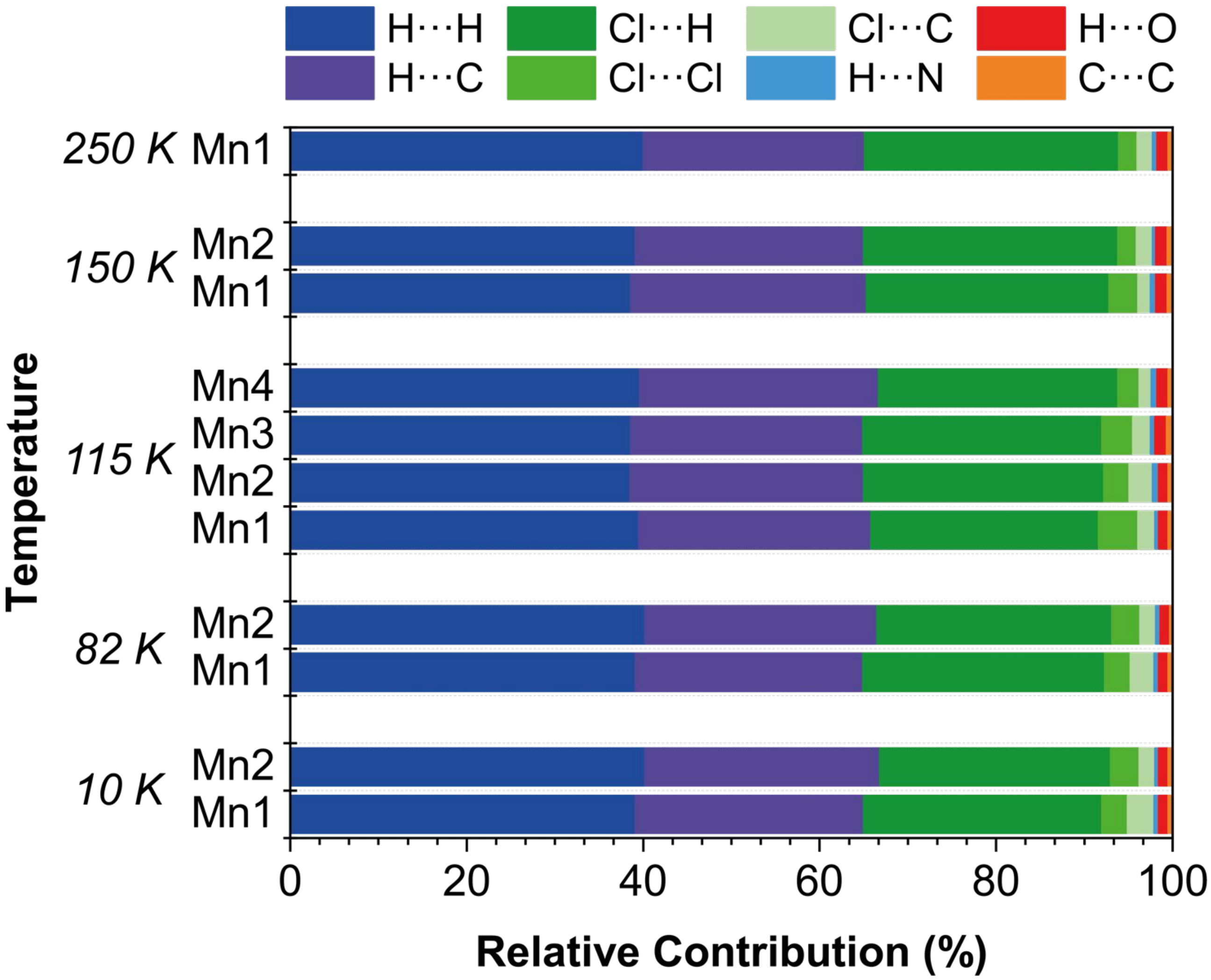

Figure S13 Relative contributions to the Hirshfeld surface area of different intermolecular interactions for the structures at 10, 82, 115, 150 and 250 K.

The dominating interactions originate from H···C, H···Cl, and H···H between the hydrogen atoms on the tetraphenylborate anion and the chloride groups on the phenyl ring on the salicylaldehyde motif as well as the hydrogen and carbon atoms in the backbone of the Mn^{3+} chelated complex **1**. Those interactions appear as distinct spikes in the 2D fingerprint plot.

S2.4 Variable temperature single crystal X-ray diffraction

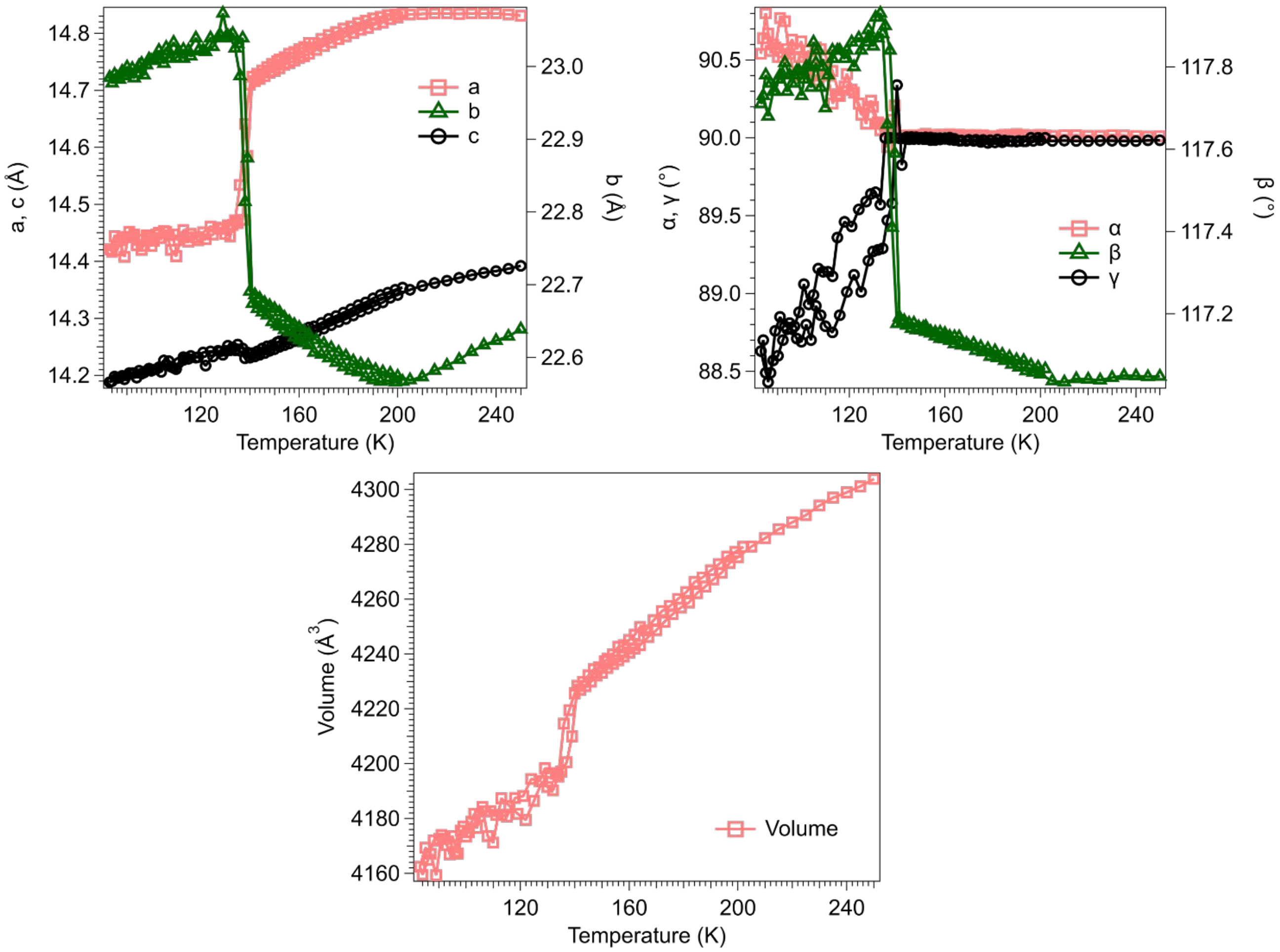

Figure S14 Variable temperature X-ray diffraction of changes in unit cell parameters, *V, a, b, c, α, β* and *γ*, measured on a single crystal of complex **1** showing the cooling and warming dependence between 250 K → 83 K → 200 K. The data was integrated with respect to the HT *Cc* cell at 250 K.

S2.5 Reconstructed planes and superstructure reflections

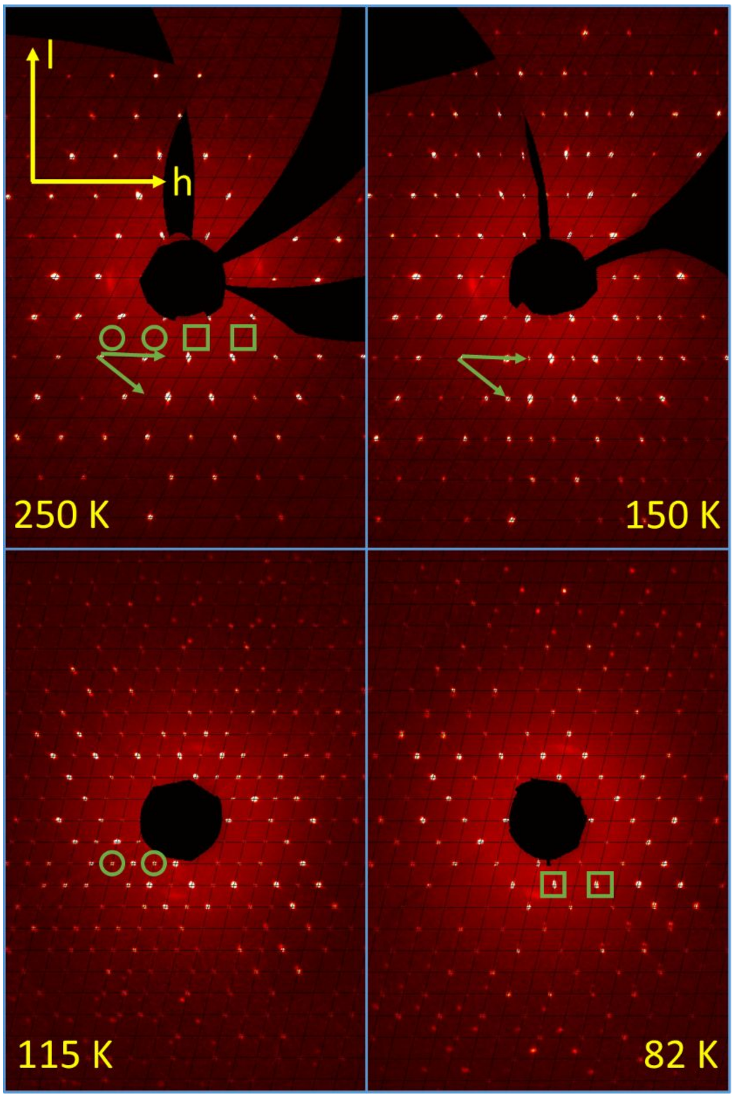

Figure S15 Representative precession images (h0l) plane obtained from variable temperature single crystal X-ray diffraction of the unit cell parameters collected on a single crystal of complex **1** at 250, 150 K, 115 K and 82 K.

S3 Resonant ultrasound spectroscopy

S3.1 Strain analysis from unit cell parameters

Spontaneous strains associated with the three structural phase transitions observed in complex **1** have been determined using the approach and equations as previously set out.[2] The parent structure has space group *Cc* and the three derivative structures have space groups *Pc*, *P1 and P1*$_{(½)}$ respectively. The group-subgroup sequences are as follows: *Cc* $\rightarrow$ *Pc* (co-elastic), *Cc* $\rightarrow$ *P1* (improper ferroelastic) and *Cc* $\rightarrow$ *P1*$_{(½)}$ (pseudoproper ferroelastic). Values of reference parameters a_o, b_o, c_o, α_o (=90°), β_o^* (= 180 - β_o), γ_o (=90°) for the *Cc* structure were obtained by extrapolation of a linear fit to measured parameters in the temperature interval 250 K to 208 K, Figure S16. A linear extrapolation does not allow for the requirement that the slope of lattice parameters of crystalline materials must tend to zero as $T \rightarrow 0$, but there is insufficient data for the *Cc* structure to allow fitting with a coth function as would normally be used for analysis of strains at low temperatures.[3-4]

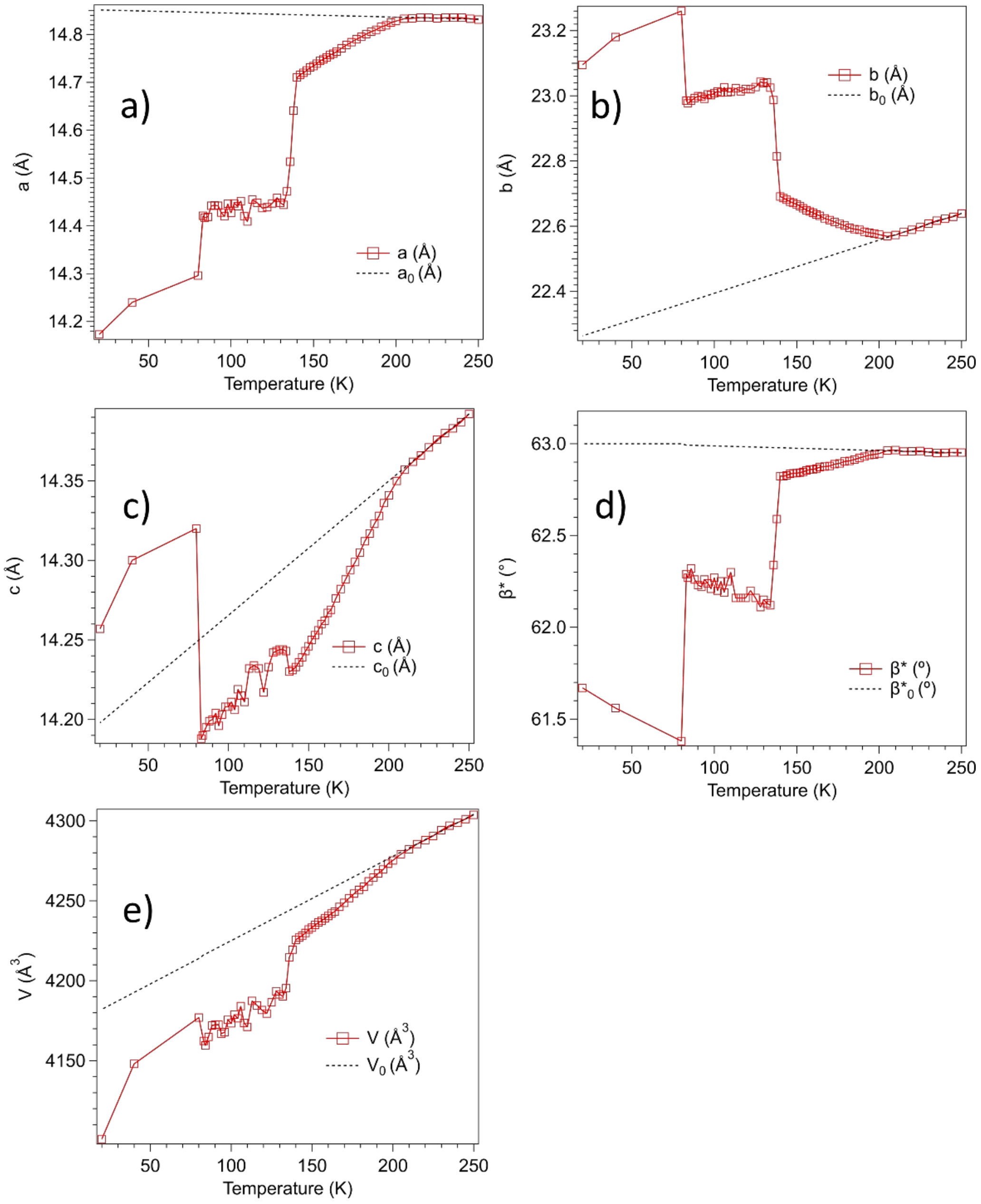

Figure S16 Linear fits to lattice parameter data in the temperature interval 250 K to 83 K. The extrapolations to lower temperatures (dotted lines) represent variations of reference parameters a_o, b_o, c_o, α_o (=90°), β_o, γ_o (=90°) in calculations of the spontaneous strains associated with *Cc* → *Pc*, *Cc* → *P1* and *Cc* → *P1*$_{(½)}$ transitions. Reference values of the unit cell volume, V_o, were obtained in the same way.

Individual strain components, e_i, i = 1, 2, 3, 5 were calculated for $Cc \rightarrow Pc$ ($e_4 = e_6 = 0$) according to the following equations:

$$e_1 = \frac{a - a_0}{a_0} \quad (S1)$$

$$e_2 = \frac{b - b_0}{b_0} \quad (S2)$$

$$e_3 = \frac{c\sin\beta - c_0\sin\beta_0}{c_0\sin\beta_0} \quad (S3)$$

$$e_5 = \left(\frac{c\cos\beta}{c_0\sin\beta_0} - \frac{a\cos\beta_0}{a_0\sin\beta_0}\right) \quad (S4)$$

Individual strain components, e_i, i = 1- 6 were calculated for $Cc \rightarrow P1$ and $Cc \rightarrow P1_{(½)}$ according to the following equations:

$$e_1 = \frac{a}{a_0}\sin(\gamma) - 1 \quad (S5)$$

$$e_2 = \frac{b}{b_0} - 1 \quad (S6)$$

$$e_3 = \frac{c\sin(\alpha)\sin(\beta^*)}{c_0\sin(\beta_0^*)} - 1 \quad (S7)$$

$$e_4 = \left(\frac{c\cos(\alpha)}{c_0\sin(\beta_0^*)} + \frac{a\cos(\beta_0^*)\cos(\gamma)}{a_0\sin(\beta_0^*)}\right) \quad (S8)$$

$$e_5 = \left(\frac{a\sin(\gamma)\cos(\beta_0^*)}{a_0\sin(\beta_0^*)} + \frac{csin(\alpha)\cos(\beta^*)}{c_0\sin(\beta_0^*)}\right) \quad (S9)$$

$$e_6 = \left(\frac{a}{a_0}\cos(\gamma)\right) \quad (S10)$$

Values of the volume strain, V_s, were given by the following equation:

$$V_s = \frac{V - V_0}{V_0} \quad (S11)$$

The resulting strain variations are given in Figures 5g,h of the main text. A test of the accuracy of linear extrapolations to obtain the reference parameters is provided by comparison of values for V_s obtained directly using Equation S11 with values obtained using $V_s = e_1 + e_2 + e_3$, which should be accurate for small volume strains. As shown in Figure 5g, the two variations of V_s have the same non-linear form of temperature dependence but with different absolute values. It is safe to conclude that the strain components all have a

continuous variation through the *Cc* → *Pc* transition point and a non-linear dependence on temperature in the stability field of the *Pc* structure. Errors in the absolute values must increase with falling temperature however, because of the assumption of linearity for the reference parameters. The *Pc* → *P1* transition is clearly discontinuous as expected, given that two space groups do not have a group-subgroup relationship. In principle it should be possible to determine the transition temperature for the *Cc* → *P1* transition by extrapolation of a fit to e_4 and e_6 to zero, but there is insufficient data to produce a reliable result.

For each of the *Cc* → *Pc* and *Cc* → *P1* transitions, the lowest order terms for coupling between strains, e_i, and the driving order parameter, q, have the form $\lambda e_i q^2$. This leads to the expected relationships $e_i \propto V_s \propto q^2$. For the *Cc* → $P1_{(½)}$ transition, the lowest order terms for coupling between strains, e_i, and the driving order parameter, q, have the form $\lambda e_i q$. This leads to the expected relationships $e_i \propto V_s \propto q$.[2]

S4 Magnetoelectric coupling

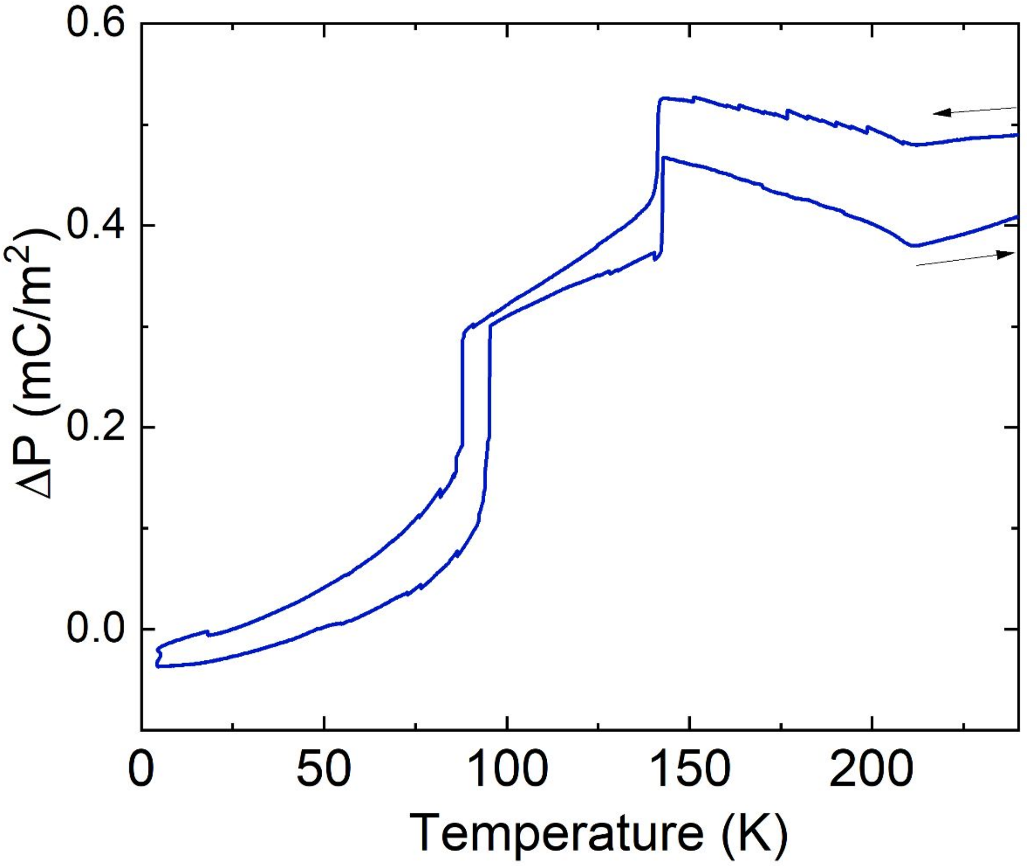

Figure S17 Electric polarization change ΔP measured vs temperature in zero externally applied electric and magnetic field for cooling and heating. The electric polarization shows sudden jumps at the LT$\rightarrow$INT1 and INT1$\rightarrow$INT2 transitions, consistent with the change in polar space group. A kink occurs at the INT2$\rightarrow$HT transition. Small discontinuous jumps and irreversibility can be attributed to the interaction of the sample with the glue that adheres it to the measurement probe.

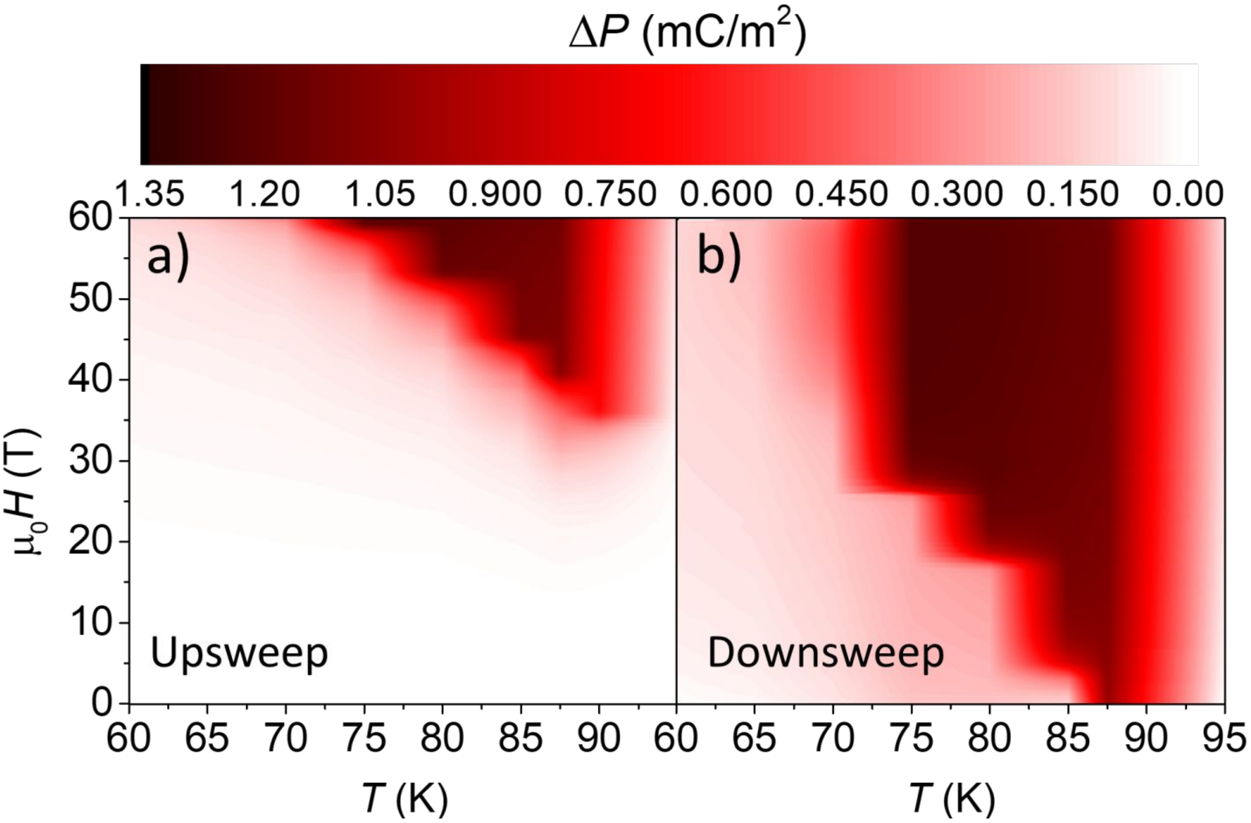

Figure S18 Electric polarization ΔP vs temperature T and magnetic field $\mu_0 H$ in a colour plot, measured in millisecond pulsed fields for up and downsweeps of the field.

S5 References

1. Turner, M. J.; McKinnon, J. J.; Wolff, S. K.; Grimwood, D. J.; Spackman, P. R.; Jayatilaka, D.; Spackman, M. A. *CrystalExplorer 17.5*, University of Western Australia: 2017.
2. Carpenter, M. A.; Salje, E. K. H.; Graeme-Barber, A., Spontaneous strain as a determinant of thermodynamic properties for phase transitions in minerals. *Eur. J. Mineral.* **1998,** *10* (4), 621-691.
3. Salje, E. K. H.; Wruck, B.; Thomas, H., Order-parameter saturation and low-temperature extension of Landau theory. *Z. Phys. B: Condens. Matter* **1991,** *82* (3), 399-404.
4. Carpenter, M. A.; Meyer, H.-W.; Sondergeld, P.; Marion, S.; Knight, K. S., Spontaneous strain variations through the low temperature phase transitions of deuterated lawsonite. *Am. Mineral.* **2003,** *88* (4), 534-546.
